# Loss of Sigma-2 Receptor/TMEM97 Is Associated with Neuropathic Injury-Induced Depression-Like Behaviors in Female Mice

**DOI:** 10.1523/ENEURO.0488-23.2024

**Published:** 2024-06-28

**Authors:** Veronica M. Hong, Avaneesh D. Rade, Shen M. Yan, Amulya Bhaskara, Muhammad Saad Yousuf, Min Chen, Stephen F. Martin, Daniel J. Liebl, Theodore J. Price, Benedict J. Kolber

**Affiliations:** ^1^Department of Neuroscience, School of Behavioral and Brain Sciences, University of Texas at Dallas, Richardson, Texas 75080; ^2^Center for Advanced Pain Studies, School of Behavioral and Brain Sciences, University of Texas at Dallas, Richardson, Texas 75080; ^3^Department of Mathematical Sciences, School of Natural Sciences and Mathematics, University of Texas at Dallas, Richardson, Texas 75080; ^4^Department of Chemistry, University of Texas at Austin, Austin, Texas 78712; ^5^Department of Neurosurgery, University of Miami, Miller School of Medicine, Miami, Florida 33146

**Keywords:** anxiety and depression, behaviors, pain, sigma-2 receptor/TMEM97

## Abstract

Previous studies have shown that ligands that bind to sigma-2 receptor/TMEM97 (s_2_R/TMEM97), a transmembrane protein, have anxiolytic/antidepressant-like properties and relieve neuropathic pain-like effects in rodents. Despite medical interest in s_2_R/TMEM97, little affective and pain behavioral characterization has been done using transgenic mice, which limits the development of s_2_R/TMEM97 as a viable therapeutic target. Using wild-type (WT) and global *Tmem97* knock-out (KO) mice, we sought to identify the contribution of *Tmem97* in modulating affective and pain-like behaviors using a battery of affective and pain assays, including open field, light/dark preference, elevated plus maze, forced swim test, tail suspension test, and the mechanical sensitivity tests. Our results demonstrate that female *Tmem97* KO mice show less anxiety-like and depressive-like behaviors in light/dark preference and tail suspension tests but not in an open field, elevated plus maze, and forced swim tests at baseline. We next performed spared nerve injury in WT and *Tmem97* KO mice to assess the role of *Tmem97* in neuropathic pain-induced anxiety and depression. WT mice, but not *Tmem97* KO mice, developed a prolonged neuropathic pain-induced depressive-like phenotype when tested 10 weeks after nerve injury in females. Our results show that *Tmem97* plays a role in modulating anxiety-like and depressive-like behaviors in naive animals with a significant change in the presence of nerve injury in female mice. Overall, these data demonstrate that *Tmem97* could be a target to alleviate affective comorbidities of pain disorders.

## Significance Statement

Chronic pain comorbidities, including anxiety and depression, present a significant public health challenge. Pharmacological agents developed to target the sigma-2 receptor/TMEM97 (s_2_R/TMEM97) have demonstrated promising effects in alleviating anxiety, depression, and pain individually. Our work provides insight on the interaction between s_2_R/TMEM97 and neuropathic pain-induced affective behaviors using transgenic mice, suggesting its potential as a novel therapeutic target for addressing both the pain and psychiatric components in complex chronic pain disorders.

## Introduction

Chronic pain comorbidities of psychiatric disorders are a major public health concern (Armbrecht et al., 2020; Johnston and Huckins, 2023); >50% of chronic pain patients suffer from psychiatric complications that aggravate pain (Banks and Kerns, 1996; Sigtermans et al., 2009; Hooten, 2016). Peripheral neuropathy is a leading chronic pain disease with psychiatric comorbidities including anxiety, depression, or both (Dersh et al., 2002; McWilliams et al., 2003; Currie and Wang, 2005; Bair et al., 2008; Agüera-Ortiz et al., 2011). The central nervous system is heavily involved in modulating pain with peripheral neuropathy (Woolf, 1996; Woolf and Mannion, 1999; Zimmermann, 2001). Due to the complex biological and cognitive manifestations of pain driven by the central nervous system (Nicholson and Verma, 2004; Bushnell et al., 2013), it is often difficult to find effective treatments for pain and associated affective comorbidities (Fishbain, 1999).

The pathogenesis of neuropathic pain-induced anxiety and depressive-like behaviors is not fully understood because most of the focus has been on neuromodulator and circuit-based investigations (S. Han et al., 2015; Zhou et al., 2019; Serafini et al., 2020; Yamauchi et al., 2022; Ji et al., 2023). The treatment of neuropathic pain with psychiatric comorbidity is experimental and unsettled. Therapies, with some efficacy, include SSRIs, SNRIs, and gabapentin (Thaler et al., 2012; Gebhardt et al., 2016; Ahmed et al., 2019). A significant limitation to all these treatments is the failure to treat both aspects of the disease (i.e., pain and affective component) at the same time leading to a potential re-occurrence of one or both disorders. There is a need to understand comorbidities and identify targets with potential efficacy for both the pain and psychiatric disease states.

One emerging target is the sigma-2 receptor/TMEM97 (s_2_R/TMEM97), a transmembrane protein located in endoplasmic reticulum membrane (Bowen et al., 1988b; Gebreselassie and Bowen, 2004; Zeng et al., 2007; Alon et al., 2017), that participates in cholesterol and calcium homeostasis (Wilcox et al., 2007; Bartz et al., 2009; Ebrahimi-Fakhari et al., 2016). In previous literature, scientists have investigated the effect of s_2_R/TMEM97 putative ligands in modulating pain (Kotagale et al., 2013; Sahn et al., 2017; Intagliata et al., 2020; Alon et al., 2021; Quadir et al., 2021; Yousuf et al., 2023) and affective states of rodents (Sánchez et al., 1997; Sánchez and Papp, 2000; Guo and Zhen, 2015). A recent paper tested the efficacy of a range of s_2_R/TMEM97 ligands in reducing neuropathic pain (Sahn et al., 2017). In this study, mice were surgically manipulated through the spared nerve injury (SNI) model to induce neuropathic pain and then tested for mechanical hypersensitivity in the context of various s_2_R/TMEM97 ligands. One of the synthetic drugs, UKH-1114, produced prolonged antinociceptive effects in a peripheral neuropathic model of mice (Sahn et al., 2017), and a subsequent study found a similar antinociceptive effects with different compounds that positively modulate the same receptor (Alon et al., 2021). s_2_R/TMEM97 has also been studied as a therapy for affective disease. In these studies, mice were tested in a series of anxiety assays, including the black and white box and the social interaction tests and depression assays such as the chronic mild stress (CMS) paradigm. It was found that siramesine HCl was comparable, if not more potent, than known clinical anxiolytic drugs, such as diazepam and lorazepam, in reversing anxiety-like behaviors in rats in these assays (Sánchez et al., 1997). Siramesine HCl also normalized sucrose solution drinking of rats to baseline in the CMS model, reversing any depressive-like effects (Sánchez and Papp, 2000). Overall, based on the literature, the modulation of s_2_R/TMEM97 found in the brain plays important role in many diseases and is associated with reduced anxiety-like and depressive-like behaviors in mice.

Although numerous putative s_2_R ligands have displayed pain-relieving, antidepressant, and anxiolytic effects, limited literature has studied the role of s_2_R/TMEM97 on interactions between pain and affective disease. In the current study, we assessed the role of s_2_R/TMEM97 in modulating neuropathic injury-induced anxiety-like and depressive-like behaviors using wild-type and global *Tmem97* knock-out mice. We used an affective behavioral battery consisting of open field, light/dark preference, elevated plus/zero maze, and forced swim and tail suspension tests before and after the neuropathic pain injury. In contrast to some existing literature, we observed that *Tmem97* disruption was protective against the development of prolonged neuropathic pain-induced depressive-like behaviors. These data highlight the value of knowing the molecular identity of s_2_R and suggest that future studies will continue to expand our knowledge of this protein.

## Materials and Methods

### Animals

All experiments used wild-type (WT) C57BL/6J (Jax000664, Jackson Laboratory) or global *Tmem97* knock-out (KO) mice (Mouse Resource & Research Centers, *Tmem97*^tm1(KOMP)Vlcg^, stock #050147-UCD). The global *Tmem97* KO mice were produced by D.J.L. (University of Miami, Florida; Vázquez-Rosa et al., 2019) and backcrossed with wild-type C57BL6/J mice in-house. A colony of these animals was bred in-house on a 12 h light/dark cycle (6 A.M.–6 P.M. lights on) and group housed with access to food and water *ad libitum*. Behavioral assays and surgeries were performed during the light cycle. Both male and female mice were at least 8 weeks old at the start of experiments. Experiments were controlled for age, sex, and littermates throughout. The experimenters were blinded to the genotype while doing surgery. Experimenters were blinded to the genotype and surgery type while performing and scoring behaviors. All experiments were in accordance with the National Institutes of Health guidelines and the institutional animal care and use committee's regulations (protocol #20-04).

### Fluorescence in situ hybridization histology

Brains from WT mice (male, *n* = 1) and *Tmem97* KO mice (male, *n* = 1) were used to perform RNAscope in situ hybridization (ISH). RNAscope ISH for *Tmem97* was performed to validate the deletion of *Tmem97* expression in *Tmem97* KO brain and to visualize *Tmem97* expression across brain sections. The brain sections that were observed are coronal sections that include the cortex, amygdala, hippocampus, and hypothalamus.

#### Sample preparation

One of 9-week-old WT (male, C57BL6/J) and one of 11-week-old *Tmem97* KO male mice were given Euthasol (10 mg/kg, i.p.). After ensuring no reflex is present by pinching toe, the vascular system was circulated with ≥30 ml of cold 1× phosphate-buffered saline (PBS), pH 7.4, and then perfused with ≥30 ml of cold 4% paraformaldehyde (PFA) solution. Brain was collected after removing skull and then postfixed in 4% PFA solution at 4°C. Brains were washed three times with cold 1× PBS and then switched to series of 10, 20, and 30% sucrose solutions for 24 h or until sunk in the sucrose solution. Then, brains were OCT-embedded in a plastic mold and frozen in a dry-iced 70% ethanol bath. OCT-embedded perfused frozen brains were equilibrated to −20°C in a cryostat and then sectioned to 20 µm thickness on a cryostat (CM 1850UV, Leica). Brain sections are directly mounted on glass slides (Superfrost, VWR) and air dried at −20°C for 2 h before storing at −80°C.

#### RNAscope in situ hybridization

The RNAscope fluorescent multiplex assay v2 kit (#323110, ACDbio) and *Tmem97* probe (#527591, ACD) were used to characterize the *Tmem97* expression as described by the manufacturer's protocol. To check the tissue quality, every batch of RNAscope experiments include at least one positive slide and one negative slide with positive probe of three control target mixture (#320861, ACD) and negative probe of no target (#320871, ACD), respectively. All perfused frozen brain sections slides were immersed and rinsed in 1× PBS, pH 7.4, for 5 min immediately after taken from −80°C. Slides then underwent each ethanol dehydration of 50%, 70%, and twice of 100% at room temperature. Slides are then incubated with hydrogen peroxide for 10 min at room temperature. After the slides were washed twice in distilled water at room temperature, slides were briefly immersed in near boiling temperature of distilled water for 10 s before fully immersed in same temperature of 1× Target Retrieval Reagent (#322000, ACD) inside the steamer (Hamilton Beach) for 5 min. Immediately after being taken out of the steamer, slides were cooled in distilled water for 15 s, immersed in 100% ethanol for 3 min and then let dry at 60°C for 5 min in the HybEZ II oven (#240200, ACD). Slides were then left at room temperature to dry out with hydrophobic barriers drawn around the brain section using ImmEdge pen (Vector Lab #H-4000). After 24 h of drying at room temperature, Protease III was gently applied to brain section and incubated for 20 min at 40°C in the humidity control tray inside the HybEZ II oven. After 20 min, slides were washed in 1× wash buffer (#310091, ACD) twice for 2 min before the probe hybridization with *Tmem97* probe (#527591, ACD), positive probe (#320881, ACD), and negative probe (#320871, ACD) for 2 h at 40°C in the humidity control tray inside the HybEZ II oven. Slides were washed in 1× washing buffer twice for 2 min each at room temperature before the series of AMP hybridization in the following order – AMP1 for 30 min, AMP2 for 30 min, and AMP3 for 15 min—at 40°C. Slides were washed twice for 2 min each in 1× wash buffer at room temperature in between each AMP steps. After the last wash, HRP signal for C1 was developed by incubating for 15 min at 40°C and washed again in 1× wash buffer. Opal dye 570 (#OP-001003, AKOYA) diluted in TSA buffer (#322809, ACD) for *Tmem97* signal was applied to brain section and incubated for 30 min at 40°C. This was followed by the same washing step with 1× wash buffer. Lastly, the HRP signal developed with Opal dye 570 (#OP-001003, AKOYA) was blocked by incubating with the HRP blocker for 15 min at 40°C. Slides with *Tmem97* probe were then immediately coverslipped with anti-fade mounting medium with DAPI (ZK0711, Vectashield). The slide with the negative probe was washed twice in 1× washing buffer for 2 min each and then counterstained with DAPI. The slide with positive probe was continued to develop HRP signals for other housekeeping gene targets with Opal dye 520 (#OP-001001, AKOYA) and Opal dye 690 (#OP-001006, AKOYA) for the C2 and C3 targets, respectively. Lastly, slides were coverslipped with anti-fade mounting medium with DAPI (ZK0711, Vectashield).

#### Image acquisition and visualization

Images were captured using an Olympus FV 3000 RS confocal laser microscope at 10× magnification with 3× zoom and stitched up for the entire brain image. All close-up brain structure images were captured at 20× magnification and other ones captured with 2× zoom and stitched up. Images used the same acquisition parameters for WT, *Tmem97* KO, and ACD negative control (data not shown) slides.

### Surgical procedure for neuropathic pain

SNI or sham surgery was performed to induce a neuropathic pain-like phenotype as described in previous studies (Lax et al., 2014; Yousuf et al., 2023). Mice were randomly assigned to the SNI versus sham group to balance the number in each group prior to the blinding. Within a single cage, half of the mice underwent sham surgery while the other half underwent SNI. Mice were briefly anesthetized in the knock-out chamber at 4% isoflurane and were under 1.5–2% isoflurane/oxygen mixture during surgery. The loss of reflex was ensured by pinching the paw with forceps and ophthalmic ointment was applied to the eyes. The hair was shaved using an electric shaver, and a small incision (<1 cm) was made using sterile scissors on the skin over the proximal location of the sciatic nerve between the knee and the hip. Under the microscope, the layers of muscle were separated using blunt scissors but with minimal tissue damage. The suture (6-0 suture) was placed underneath the tibial and common peroneal branches of the nerve. These two branches of the nerve were ligated with sutures with two tight surgical knots on either side of the knee. A nerve segment in between two surgical knots was cut out using microscissors to prevent nerve regeneration. Forceps were used to hold the ligated nerve while cutting to leave the sural nerve completely intact. The sham surgery was done in the same manner with no nerve manipulation. The skin was closed and sutured with surgical knots. A single dose of 10% gentamicin solution at 100 µl (Sigma Aldrich #G1272) was subcutaneously injected to prevent infections. Mice were placed back in home cages with gel supplements (DietGel, ClearH2O) and a heating pad underneath the cage to recover from acute postoperative pain. Mice were checked every other day for any infection or loosened sutures for up to 2 weeks.

### Behavioral testing

#### Experimental procedure

A series of behavioral tests were conducted on both WT and *Tmem97* KO mice to evaluate anxiety-like and depressive-like behaviors. These affective behavior tests included open field (OF), light/dark preference (LDP), elevated plus maze (EPM), elevated zero maze (EZM), forced swim test (FST), and tail suspension test (TST), followed by sensorimotor tests. There was a 2–3 d interval between each assay and the order of the behaviors was consistent across all cohorts of mice as illustrated (Fig. 2). Two to three days following the last baseline behavioral test, mechanical hypersensitivity was measured using the von Frey method for 2 consecutive days to establish a baseline sensitivity measurement before injury. A subset of mice that underwent SNI surgery (or sham treatment) were then tested for mechanical hypersensitivity weekly for 4 weeks postinjury. After 10–14 weeks post-SNI surgery (or sham surgery), affective behavior tests were repeated in the same manner as before the injury (Fig. 6).

Testing order of mice was created by assigning a randomized letter per animal (e.g., A to Z) using a randomized function in Excel. For behavioral testing, the order of testing for mice was kept consistent across all behaviors (e.g., A, then B, then C, etc.), and testing was completed blinded to genotype and surgery (if applicable). All experiments were done with four cohorts of mice in total and each cohort included WT and *Tmem97* KO mice in both sexes with matched ages within and across cohorts. The first cohort of mice were not tested on elevated plus maze, elevated zero maze, von Frey mechanical sensitivity testing, and the sensorimotor test, but all other cohorts went through the entire behavioral battery.

#### Open field

Mice were habituated in the room with 60–70 dB of white noise and under bright light (4,400–4,500 lux) for ∼1 h with the experimenter in the room for the last 30 min prior to the testing. Anxiety and locomotor activity were measured using open-field testing (Seibenhener and Wooten, 2015). The open-field apparatus was custom-made with a white Plexiglass box (25 × 25 × 35 cm). Mice were gently placed in the top and right corner of the open-field apparatus and were allowed to freely move inside the box for a total of 20 min. ANY-maze video tracking system (Stoelting) connected with an overhead camera was used to record the video and analyze the data. The inside zone was defined by the 12.5 × 12.5 cm zone in the center of the open-field apparatus, and the outside zone is defined by any space outside the center. The time spent and distance traveled were measured in the outside and inside zone.

#### Light/dark preference

Anxiety-like behavior was measured using the light/dark preference (Bourin and Hascoët, 2003; Lax et al., 2018). Mice were habituated in the same manner as open-field testing. The Plexiglass light/dark preference apparatus is divided into open lid white (27.5 × 27 cm) and closed lid black (18.5 × 27 cm) compartments, which serve as light and dark sides, respectively. The sliding door in between the white and black compartments opens a 5 × 6 cm size door for mice to move in between the compartments. Mice were placed in the closed black compartment in the complete dark. After 1 min on the dark side, the sliding door is opened to allow mice to move freely between the light and dark sides for a total of 10 min (Kolber et al., 2010; Lax et al., 2018). Using the ANY-Maze video tracking system, the following variables were measured: number of entries to the light side, time spent on the light side, average time spent for each entry, and latency to enter the light side.

#### Elevated plus maze and elevated zero maze

Anxiety-like behavior was measured using the elevated plus maze apparatus and elevated zero maze apparatus (Kulkarni et al., 2007). Mice were habituated in the same manner as open-field testing under the ceiling light. The elevated plus maze is a cross-shaped maze with two open arms (36 × 6 cm) and two closed arms (36 × 6 × 17 cm) with black opaque walls that intersect each other in the center (6 × 6 cm). The elevated zero maze apparatus (Stoelting) is a circle-shaped maze (50 cm in diameter) with two open-arm portions (5 × 1.3 cm) and two closed-arm portions with an opaque wall (5 × 15 cm). Both mazes were 61 cm off the ground and isolated from the rest of the room with a black curtain drawn around the apparatus to block context cues. Mice were gently placed on the end of the closed arm/portion and allowed to move freely for a total of 10 min (Lax et al., 2018). If they fell, the recording was paused and resumed after placing them in their position prior to falling. The following variables were measured: the time spent, and the distance traveled in the closed arm and open arm.

#### Forced swim test

Depressive-like behavior was measured using the forced swim test (Can et al., 2012a). Mice were habituated in the same manner as open-field testing but with less bright ceiling light (2,000 lux). A glass beaker (1 L) was filled up with ∼750 ml of water at ∼30°C and placed on the table with the overhead web camera (Logitech) to live record. After habituation, the mouse was gently placed in the beaker of water for a total of 6–7 min (Kolber et al., 2010; Lax et al., 2018). The immobility time was measured in first six 1 min bins. The mobile movements of mice were defined by anything other than floating, including one foot gently moving, paddling with hindpaws, swishing back and forth, and touching glass walls with a forepaw. After testing, mice were placed in an empty cage with a heat lamp overhead to warm mice. Then, mice were transferred back to their home cages.

#### Tail suspension test

Depressive-like behaviors were measured using the tail suspension test (Can et al., 2012b). Mice were habituated in the same manner as the forced swim test. Mice were suspended upside down with the end of their tail taped on a platform clamped to a white Plexiglass (25 × 25 × 35 cm) box with their ventral side facing the camera and experimenter. A small cylindrical plastic tube was fitted on to the tail base to prevent mice from grabbing their tails. The movement of each animal was recorded for a total of 6–7 min (Kolber et al., 2010; Lax et al., 2018). The immobility time was measured in first six 1 min bins. The mobile movements of mice were defined as any extraneous movement that caused the mouse to swing laterally, movement of the hindpaws and forepaws, and using the forepaws to grab one or both hindpaws.

#### Marble burying

Marble burying was tested as described in previous studies (Deacon, 2006; Angoa-Pérez et al., 2013) with modification. Prior to testing, mice were handled by experimenters for 2 min each for 2 consecutive days prior to initiating the assay. On the day of testing, mice were habituated for 1 h in their home cages in the behavior testing room with ceiling lights (2,000 lux) and speakers playing white noise (∼64 dB). The marbles, uniform in size and shape but varying in color, were placed in a 3 × 5 grid-like fashion within the cage. The marbles were set up in new cages that had enough bedding to create a 1-inch-thick layer. After habituation, the mice were placed into the cage and allowed to freely explore for 30 min. Once the trial had elapsed, the mice were transferred back to their cages, and the number of marbles that had ∼2/3 of their surface area covered by bedding was manually counted. After counting, the bedding was changed out and the marbles were cleaned with 70% ethanol and dried before replacing them for the next trial.

#### Nestlet shredding

Nestlet shredding was tested as described (Angoa-Pérez et al., 2013) with modification. Mice were handled and habituated in the same manner as for marble burying. A cotton nestlet was placed in a new cage that had enough bedding to cover the transparent bottom of the cage. Each cage had a square cotton nestlet (5 × 5 cm) placed in the center, equidistant from the walls of the cage. Prior to testing, each nestlet was weighed to determine its mass. After habituation, the mice were placed into the cage and allowed to freely explore for 30 min. Once the trial had elapsed, the mice were transferred back to their home cages, and the nestlets were allowed to dry in the same behavior room overnight. The following day, the nestlets were weighed again and the percent change in mass was calculated. The bedding was changed out to new bedding and new nestlets were placed in the test cages.

#### Novel object recognition

Novel object recognition was tested as described in previous studies (Leger et al., 2013; Lueptow, 2017) with modification. Prior to testing, mice were handled by experimenters for 2 min each for 2 consecutive days prior to initiating the assay. On the day of testing, mice were habituated for 1 h in their home cages in the behavior testing room with ceiling lights (2,000 lux) and speakers playing white noise (∼64 dB). The novel object recognition box (25 × 25 × 35 cm) is a Plexiglass box with opaque side walls and an open top. The objects are made of painted wood and are distinct in shape and color. The behavior paradigm consisted of a training phase and a testing phase. During the training phase, two identical objects were placed in the box 5 cm from each wall in adjacent corners. Mice were placed into the box and allowed to explore freely for 10 min, with an overhead camera live recording their behavior. Then, the mice were transferred back to their home cages. During the testing phase, one of the familiar objects was randomly selected to remain in the box, and the novel object, distinct in shape and color, was placed equidistant from all walls (as noted in the training phase) in the adjacent corner, replacing the familiar object that was removed. Then, 1 h after the completion of the training phase, mice were placed into the box and allowed to freely explore the new environment for 10 min, with an overhead camera live recording their behavior, including time spent with each object and the number of investigations for each object. Then, the mice were transferred back to their cages. The recognition memory of the mice was analyzed by calculating the discrimination index for time spent with the object and number of investigations of the object:

$$\mathrm{Discrimination}\;\mathrm{Index}\;(\mathrm{Time})=\frac{\mathrm{Time}\;\;\mathrm{with}\;\mathrm{Novel}\;\mathrm{Object}-\mathrm{Time}\;\mathrm{with}\;\mathrm{Familiar}\;\mathrm{Object}}{\mathrm{Total}\;\mathrm{Exploration}\;\mathrm{Time}},$$

$$\mathrm{Discrimination}\;\mathrm{Index}\;(\#\;\mathrm{of}\;\mathrm{Investigations})=\;\frac{\#\;\mathrm{of}\;\;\mathrm{Investigations}\;\mathrm{of}\;\mathrm{Novel}\;\mathrm{Object}-\;\#\;\mathrm{of}\;\mathrm{Investigations}\;\mathrm{of}\;\mathrm{Familiar}\;\mathrm{Object}}{\mathrm{Total}\;\#\;\mathrm{of}\;\mathrm{investigations}}.$$

A positive discrimination index indicated mice preferred the novel object, while a negative discrimination index indicated mice preferred the familiar object (Antunes and Biala, 2012).

#### Novel object location

Novel object location was tested as described in previous studies (Denninger et al., 2018) with modification. Mice were handled and habituated in the same manner as described for novel object recognition. The testing box and objects used for novel object recognition were also used for novel object location. The behavior paradigm consists of a training phase and a testing phase. During the training phase, two identical objects were placed in the box 5 cm from each wall, in adjacent corners. Mice were placed into the box and allowed to explore freely for 10 min, with an overhead camera live recording their behavior. Then, the mice were transferred back to their home cages. During the testing phase, one of the familiar objects was randomly selected to remain in the box in its position, and the other familiar object was placed equidistant from all walls (as noted in the training phase) in the opposite corner from the object that was not moved. Then, 1 h after the completion of the training phase, mice were placed into the box and allowed to freely explore the new environment for 10 min, with an overhead camera live recording their behavior, including time spent with each object and the number of investigations for each object. The mice were transferred back to their home cages. The location memory of the mice was tested by calculating the discrimination index for time spent with the objects and number of investigations of the objects, using the same formulas as described for novel object recognition. A positive discrimination index indicated mice preferred the displaced object, while a negative discrimination index indicated mice preferred the object that was not displaced.

#### von Frey mechanical sensitivity

Mice were placed in a Plexiglass enclosure (10 × 10 × 15 cm) covered with black cardboard on wire mesh. Mice were habituated with the 60–70 dB of white noise for ∼2 h with the experimenter in the room for the last 30 min prior to the testing. Withdrawal thresholds were measured on the lateral side of both paws. The calibrated von Frey filaments of 0.02, 0.04, 0.08, 0.16, 0.32, 0.64, 1.28, and 2.56 g (0.2, 0.4, 0.8, 1.6, 3.2, 6.4, 12.8, 25.6 mN) were used, and the results were analyzed using the up-down assessment method as previously described in other studies (Dixon, 1965; Chaplan et al., 1994; Sadler et al., 2017). The two trials for each paw were performed and averaged for 50% withdrawal threshold for each paw. Mechanical hypersensitivity was measured every week for 4 weeks.

#### Sensorimotor test

Mice were evaluated on their general sensorimotor batteries to screen any motor function deficits. Tests were conducted before and after the SNI surgery as described previously (Jain et al., 2006; Golden et al., 2010; Kolber et al., 2010) to evaluate balance, strength, coordination, and movement. These tests consist of walk initiation, turn-on-pole, ledge, platform, 60° and 90° inclined wire mesh, and the inverted wire mesh. The walk initiation was evaluated based on the time the mice took to leave the square shaped area (21 × 21 cm) marked by tape. For the turn-on-pole test, mice were allowed to grasp onto the top of the pole with their front paws and face upward by dangling their heads nearby the pole while holding on with their tails. The time it took to fully turn their body orientation downward and the time it took come down to the bottom of the pole without falling were measured. The vertical pole (55 cm) had no platform and had grooves for mice to grasp. The ledge and platform tests were evaluated based on how long mice could maintain their balance on an edge or platform without falling. Mice were allowed to balance on a narrow Plexiglass ledge (0.75 cm thick) for the ledge test or on an elevated circular platform (3 cm diameter × 1 cm thick, 47 cm above the floor) for the platform test. Lastly, mice were placed on an inclined wire mesh (60° first and then 90°) oriented downward, and the time it took before reaching either end of the mesh was measured. For the inverted mesh test, the wire mesh was flipped 180° right after mice were placed on at 90° and then the latency to fall was measured. A maximum of 60 s was allowed for walk initiation, ledge, platform, and 60° or 90° inclined wire mesh test. A maximum of 120 s was allowed for turn-on-pole and inverted wire mesh test. Every test had two trials except for the inverted wire mesh test, in which mice quickly learned to fall safely from the first trial.

### Statistical and data analysis

All data analyses were initially conducted using Prism (GraphPad V10) followed by SAS (SAS Institute, STAT 15.1) for data requiring *a posteriori* variable selection statistical modeling of *post hoc* analyses. All graphs were generated in Prism. For two-factor analyses, behavioral data were analyzed using two-way ANOVAs followed by Tukey's multiple-comparisons tests on comparisons that were significant for main effects/interactions. For analysis of mechanical sensitivity data, a two-way ANOVA was completed with two factors (group and time) followed by Tukey's multiple-comparisons tests to compare genotypes and treatments at each time point. For three-factor analyses of sex/genotype/treatment (Fig. 7), we detected unequal variances and adopted a variable selection three-way ANOVA model adjusted for different variances and *post hoc* Bonferroni’s analysis on significant effects only. For sensory motor test analysis of genotype and treatment with time as a repeated variable (Extended Data Fig. 8-1), we completed a repeated-measures mixed model (three-way ANOVA) with Bonferroni’s multiple-comparisons tests only for significant interaction effects.

Asterisks denote *p* values in the following manner: *p < 0.05, **p < 0.01, ***p < 0.001, and ****p < 0.0001. All data was analyzed as the mean ± SEM. Statistical information for all figures is summarized in Table 1.

**Table 1.** Summary of statistical information for all figures

Figure	Comparison	Analysis	*p* value, *F*, or *t* value	*N*
3*A*	Open field—total distance traveled by genotype	Two-way ANOVA with Tukey's multiple-comparisons test	Sex: *p* = 0.6430; *F*_(1,68)_ = 0.22 Genotype: *p* = 0.8755; *F*_(1,68)_ = 0.025 Interaction: *p* = 0.1968; *F*_(1,68)_ = 1.70	Male WT = 18 Female WT = 15 Male *Tmem97* KO = 20 Female *Tmem97* KO = 19
3*B*	Open field—time spent in center zone by genotype	Two-way ANOVA with Tukey's multiple-comparisons test	Sex: ****p < 0.0001; *F*_(1,68)_ = 35.04 Genotype: *p* = 0.2163; *F*_(1,68)_ = 1.56 Interaction: *p* = 0.0918; *F*_(1,68)_ = 2.92 Sex effect Male WT vs female WT: ****p < 0.0001 Male KO vs female KO: *p = 0.0160	Male WT = 18 Female WT = 15 Male *Tmem97* KO = 20 Female *Tmem97* KO = 19
3*C*	Open field—distance traveled in center zone by genotype	Two-way ANOVA with Tukey's multiple-comparisons test	Sex: *p* = 0.6736; *F*_(1,68)_ = 0.18 Genotype: *p* = 0.8806; *F*_(1,68)_ = 0.023 Interaction: *p* = 0.4798; *F*_(1,68)_ = 0.50	Male WT = 18 Female WT = 15 Male *Tmem97* KO = 20 Female *Tmem97* KO = 19
3*D*	Light/dark preference—number of entries to light side by genotype	Two-way ANOVA with Tukey's multiple-comparisons test	Sex: *p* = 0.1888; *F*_(1,68)_ = 1.76 Genotype: *p* = 0.0612; *F*_(1,68)_ = 3.62 Interaction: *p* = 0.2503; *F*_(1,68)_ = 1.34	Male WT = 18 Female WT = 15 Male *Tmem97* KO = 20 Female *Tmem97* KO = 19
3*E*	Light/dark preference—time spent in light side by genotype	Two-way ANOVA with Tukey's multiple-comparisons test	Sex: *p* = 0.0589; *F*_(1,68)_ = 3.69 Genotype: *p = 0.0205; *F*_(1,68)_ = 5.63 Interaction: *p* = 0.1534; *F*_(1,68)_ = 2.08 Genotype effect Male WT vs male KO: *p* = 0.9053 Female WT vs female KO: *p* = 0.0518	Male WT = 18 Female WT = 15 Male *Tmem97* KO = 20 Female *Tmem97* KO = 19
3*F*	Light/dark preference—average time spent per visit in light side by genotype	Two-way ANOVA with Tukey's multiple-comparisons test	Sex: *p = 0.0137; *F*_(1,68)_ = 6.40 Genotype: **p = 0.0097; *F*_(1,68)_ = 7.08 Interaction: *p* = 0.1752; *F*_(1,68)_ = 1.88 Sex effect Male WT vs female WT: *p = 0.0487 Male KO vs female KO: *p* = 0.8262 Genotype effect Male WT vs male KO: *p* = 0.7827 Female WT vs female KO: *p = 0.0358	Male WT = 18 Female WT = 15 Male *Tmem97* KO = 20 Female *Tmem97* KO = 19
3*G*	Light/dark preference—latency to first entry to light side by genotype	Two-way ANOVA with Tukey's multiple-comparisons test	Sex: *p* = 0.7564; *F*_(1,68)_ = 0.097 Genotype: *p* = 0.5405; *F*_(1,68)_ = 0.38 Interaction: *p* = 0.2777; *F*_(1,68)_ = 1.20	Male WT = 18 Female WT = 15 Male *Tmem97* KO = 20 Female *Tmem97* KO = 19
3*H*	Elevated plus maze—total distance traveled by genotype	Two-way ANOVA with Tukey's multiple-comparisons test	Sex: *p = 0.0421; *F*_(1,51)_ = 4.35 Genotype: *p* = 0.2287; *F*_(1,51)_ = 1.48 Interaction: *p* = 0.0773; *F*_(1,51)_ = 3.25 Sex effect Male WT vs female WT: *p* = 0.0635 Male KO vs female KO: *p* = 0.9964	Male WT = 15 Female WT = 9 Male *Tmem97* KO = 16 Female *Tmem97* KO = 15
3*I*	Elevated plus maze—number of entries in open arm by genotype	Two-way ANOVA with Tukey's multiple-comparisons test	Sex: *p* = 0.4933; *F*_(1,51)_ = 0.48 Genotype: *p* = 0.6114; *F*_(1,51)_ = 0.26 Interaction: *p* = 0.1682; *F*_(1,51)_ = 1.95	Male WT = 15 Female WT = 9 Male *Tmem97* KO = 16 Female *Tmem97* KO = 15
3*J*	Elevated plus maze—time spent in open arm by genotype	Two-way ANOVA with Tukey's multiple-comparisons test	Sex: *p* = 0.8844; *F*_(1,51)_ = 0.021 Genotype: *p* = 0.3175; *F*_(1,51)_ = 1.02 Interaction: *p* = 0.6882; *F*_(1,51)_ = 0.16	Male WT = 15 Female WT = 9 Male *Tmem97* KO = 16 Female *Tmem97* KO = 15
3*K*	Forced swim test—duration of immobility by genotype	Two-way ANOVA with Tukey's multiple-comparisons test	Sex: *p* = 0.8739; *F*_(1,68)_ = 0.025 Genotype: *p* = 0.3476; *F*_(1,68)_ = 0.89 Interaction: *p* = 0.3526; *F*_(1,68)_ = 0.88	Male WT = 18 Female WT = 15 Male *Tmem97* KO = 20 Female *Tmem97* KO = 19
3*L*	Tail suspension test—duration of immobility by genotype	Two-way ANOVA with Tukey's multiple-comparisons test	Sex: *p* = 0.7254; *F*_(1,68)_ = 0.12 Genotype: **p = 0.0013; *F*_(1,68)_ = 11.24 Interaction: *p* = 0.1799; *F*_(1,68)_ = 1.84 Genotype effect Male WT vs male KO: *p* = 0.4683 Female WT vs female KO: *p = 0.0100	Male WT = 18 Female WT = 15 Male *Tmem97* KO = 20 Female *Tmem97* KO = 19
4*A*	*Z* score on open field by genotype and sex	Two-way ANOVA with Tukey's multiple-comparisons test	Sex: *p* = 0.6953; *F*_(1,68)_ = 0.15 Genotype: *p* = 0.6787; *F*_(1,68)_ = 0.17 Interaction: *p* = 0.6480; *F*_(1,68)_ = 0.21	Male WT = 18 Female WT = 15 Male *Tmem97* KO = 20 Female *Tmem97* KO = 19
4*B*	*Z* score on light/dark preference	Two-way ANOVA with Tukey's multiple-comparisons test	Sex: *p* = 0.0509; *F*_(1,68)_ = 3.95 Genotype: *p = 0.0106; *F*_(1,68)_ = 6.90 Interaction: *p* = 0.0944; *F*_(1,68)_ = 0.094 Genotype effect Male WT vs male KO: *p* = 0.9045 Female WT vs female KO: *p = 0.0210	Male WT = 18 Female WT = 15 Male *Tmem97* KO = 20 Female *Tmem97* KO = 19
4*C*	*Z* score on elevated plus maze	Two-way ANOVA with Tukey's multiple-comparisons test	Sex: *p* = 0.6683; *F*_(1,51)_ = 0.19 Genotype: *p* = 0.4270; *F*_(1,51)_ = 0.64 Interaction: *p* = 0.3565; *F*_(1,51)_ = 0.87	Male WT = 15 Female WT = 9 Male *Tmem97* KO = 16 Female *Tmem97* KO = 15
4*D*	*Z* score on elevated zero maze	Two-way ANOVA with Tukey's multiple-comparisons test	Sex: *p* = 0.1956; *F*_(1,51)_ = 1.72 Genotype: *p* = 0.5834; *F*_(1,51)_ = 0.30 Interaction: *p* = 0.5895; *F*_(1,51)_ = 0.29	Male WT = 15 Female WT = 9 Male *Tmem97* KO = 16 Female *Tmem97* KO = 15
4*E*	*Z* score on forced swim test	Two-way ANOVA with Tukey's multiple-comparisons test	Sex: *p* = 0.8752; *F*_(1,68)_ = 0.025 Genotype: *p* = 0.3549; *F*_(1,68)_ = 0.87 Interaction: *p* = 0.3454; *F*_(1,68)_ = 0.9028	Male WT = 18 Female WT = 15 Male *Tmem97* KO = 20 Female *Tmem97* KO = 19
4*F*	*Z* score on tail suspension test	Two-way ANOVA with Tukey's multiple-comparisons test	Sex: *p* = 0.7254; *F*_(1,68)_ = 0.12 Genotype: **p = 0.0013; *F*_(1,68)_ = 11.24 Interaction: *p* = 0.1799; *F*_(1,68)_ = 1.84 Genotype effect Male WT vs male KO: *p* = 0.4683 Female WT vs female KO: *p = 0.0100	Male WT = 18 Female WT = 15 Male *Tmem97* KO = 20 Female *Tmem97* KO = 19
4*G*	Overall *Z* score on emotionality	Two-way ANOVA with Tukey's multiple-comparisons test	Sex: *p* = 0.9888; *F*_(1,68)_ = 0.00020 Genotype: ***p = 0.0008; *F*_(1,68)_ = 12.37 Interaction: *p* = 0.8085; *F*_(1,68)_ = 0.059 Genotype effect Male WT vs male KO: *p* = 0.0889 Female WT vs female KO: *p* = 0.0568	Male WT = 15–18 Female WT = 9–15 Male *Tmem97* KO = 16–20 Female *Tmem97* KO = 15–19
5*B*	Nestlet shredding—percent mass change	Two-way ANOVA with Tukey's multiple-comparisons test	Sex: *p* = 0.6288; *F*_(1,30)_ = 0.24 Genotype: *p* = 0.9455; *F*_(1,30)_ = 0.0048 Interaction: *p* = 0.4405; *F*_(1,30)_ = 0.61	Male WT = 11 Female WT = 5 Male *Tmem97* KO = 12 Female *Tmem97* KO = 6
5*C*	Marble burying—number of marbles buried	Two-way ANOVA with Tukey's multiple-comparisons test	Sex: *p* = 0.0651; *F*_(1,30)_ = 3.67 Genotype: *p* = 0.8778; *F*_(1,30)_ = 0.024 Interaction: *p* = 0.1093; *F*_(1,30)_ = 2.72	Male WT = 11 Female WT = 5 Male *Tmem97* KO = 11 Female *Tmem97* KO = 6
5*D*	Novel object recognition for time investigated—relative discrimination index	Two-way ANOVA with Tukey's multiple-comparisons test	Sex: *p* = 0.9651; *F*_(1,26)_ = 0.0019 Genotype: *p* = 0.5186; *F*_(1,26)_ = 0.43 Interaction: *p* = 0.3282; *F*_(1,26)_ = 0.99	Male WT = 11 Female WT = 5 Male *Tmem97* KO = 8 Female *Tmem97* KO = 6
5*E*	Novel object location for number of entries investigated—relative discrimination index	Two-way ANOVA with Tukey's multiple-comparisons test	Sex: *p* = 0.4110; *F*_(1,26)_ = 0.70 Genotype: *p* = 0.8019; *F*_(1,26)_ = 0.064 Interaction: *p* = 0.6409; *F*_(1,26)_ = 0.22	Male WT = 11 Female WT = 5 Male *Tmem97* KO = 8 Female *Tmem97* KO = 6
7*A*	Open field—total distance traveled at pre- and postsurgery	Three-way ANOVA with allowing different variances for genotype	Treatment: *p* = 0.9523; *F*_(1,64)_ = 0.0036 Sex: *p* = 0.1051; *F*_(1,64)_ = 2.7 Genotype: *p* = 0.3333; *F*_(1,64)_ = 0.95 Treatment × sex: *p* = 0.2051; *F*_(1,64)_ = 1.64 Treatment × genotype: *p* = 0.4616; *F*_(1,64)_ = 0.55 Sex × genotype: *p* = 0.2563; *F*_(1,64)_ = 1.31 Treatment × sex × genotype: *p* = 0.8964; *F*_(1,64)_ = 0.017	Male WT SNI = 9 Male WT sham = 9 Male KO SNI = 10 Male KO sham = 10 Female WT SNI = 9 Female WT sham = 6 Female KO SNI = 9 Female KO sham = 10
7*B*	Open field—distance traveled in center at pre- and postsurgery	Three-way ANOVA with allowing different variances for genotype	Treatment: *p* = 0.4413; *F*_(1,64)_ = 0.60 Sex: *p* = 0.6033; *F*_(1,64)_ = 0.27 Genotype: *p* = 0.1211; *F*_(1,64)_ = 2.47 Treatment × sex: *p* = 0.2860; *F*_(1,64)_ = 1.16 Treatment × genotype: *p* = 0.8159; *F*_(1,64)_ = 0.055 Sex × genotype: *p* = 0.1890; *F*_(1,64)_ = 1.76 Treatment × sex × genotype: *p* = 0.4828; *F*_(1,64)_ = 0.50	Male WT SNI = 9 Male WT sham = 9 Male KO SNI = 10 Male KO sham = 10 Female WT SNI = 9 Female WT sham = 6 Female KO SNI = 9 Female KO sham = 10
7*C*	Open field—time spent in center at pre- and postsurgery	Three-way ANOVA with allowing different variances for genotype	Treatment: *p* = 0.6740; *F*_(1,64)_ = 0.18 Sex: *p* = 0.2267; *F*_(1,64)_ = 1.49 Genotype: *p* = 0.2688; *F*_(1,64)_ = 1.24 Treatment × sex: *p* = 0.5807; *F*_(1,64)_ = 0.31 Treatment × genotype: *p* = 0.2760; *F*_(1,64)_ = 1.21 Sex × genotype: *p* = 0.2691; *F*_(1,64)_ = 1.24 Treatment × sex × genotype: *p* = 0.1349; *F*_(1,64)_ = 2.29	Male WT SNI = 9 Male WT sham = 9 Male KO SNI = 10 Male KO sham = 10 Female WT SNI = 9 Female WT sham = 6 Female KO SNI = 9 Female KO sham = 10
7*D*	Light/dark preference—number of entries to light side at pre- and postsurgery	Three-way ANOVA with allowing different variances for genotype and adjusted for Bonferroni’s method (nonsignificant interaction effect dropped for post hoc purpose)	Treatment: *p* = 0.1393; *F*_(1,65.4)_ = 2.24 Sex: *p = 0.0235; *F*_(1,62)_ = 5.40 Genotype: *p* = 0.1323; *F*_(1,62.1)_ = 2.33 Sex × treatment: *p = 0.0300; *F*_(1,65.4)_ = 4.92 Genotype × sex: *p = 0.0227; *F*_(1,62.1)_ = 5.46 Sex × treatment effect: adjusted *p* value Female SNI vs female sham: *p* = 0.9599 Female SNI vs male SNI: *p* = 0.9997 Female SNI vs male sham: *p = 0.0369 Female sham vs male SNI: *p* = 0.9350 Female sham vs male sham: *p = 0.0128 Male SNI vs male sham: *p = 0.0415 Genotype × sex effect: adjusted *p* value KO female vs KO male: *p* = 1.000 KO female vs WT female: *p* = 0.0502 KO female vs WT male: *p* = 0.9394 KO male vs WT female: *p = 0.0478 KO male vs WT male: *p* = 0.9329 WT female vs WT male: *p = 0.0177 Significant post hoc sex effects Sham WT male vs sham WT female: *p = 0.0011 SNI WT male vs SNI WT female: *p* = 0.3324	Male WT SNI = 9 Male WT sham = 9 Male KO SNI = 10 Male KO sham = 10 Female WT SNI = 9 Female WT sham = 6 Female KO SNI = 9 Female KO sham = 10
7*E*	Light/dark preference—time spent in light side at pre- and postsurgery	Three-way ANOVA with allowing different variances for genotype	Treatment: *p* = 0.6966; *F*_(1,64)_ = 0.15 Sex: *p* = 0.1308; *F*_(1,64)_ = 2.34 Genotype: *p* = 0.4829; *F*_(1,64)_ = 0.50 Treatment × sex: *p* = 0.3496; *F*_(1,64)_ = 0.89 Treatment × genotype: *p* = 0.7381; *F*_(1,64)_ = 0.11 Sex × genotype: *p* = 0.2320; *F*_(1,64)_ = 1.46 Treatment × sex × genotype: *p* = 0.4074; *F*_(1,64)_ = 0.70	Male WT SNI = 9 Male WT sham = 9 Male KO SNI = 10 Male KO sham = 10 Female WT SNI = 9 Female WT sham = 6 Female KO SNI = 9 Female KO sham = 10
7*F*	Light/dark preference—average time spent per visit in light side at pre- and postsurgery	Three-way ANOVA with allowing different variances for genotype	Treatment: *p* = 0.6658; *F*_(1,64)_ = 0.19 Sex: *p* = 0.8027; *F*_(1,64)_ = 0.063 Genotype: *p* = 0.5007; *F*_(1,64)_ = 0.46 Treatment × sex: *p* = 0.9823; *F*_(1,64)_ = 0.00050 Treatment × genotype: *p* = 0.5690; *F*_(1,64)_ = 0.33 Sex × genotype: *p* = 0.7305; *F*_(1,64)_ = 0.12 Treatment × sex × genotype: *p* = 0.3753; *F*_(1,64)_ = 0.80	Male WT SNI = 9 Male WT sham = 9 Male KO SNI = 10 Male KO sham = 10 Female WT SNI = 9 Female WT sham = 6 Female KO SNI = 9 Female KO sham = 10
7*G*	Light/dark preference—latency to first entry to light side at pre- and postsurgery	Three-way ANOVA with allowing different variances for genotype	Treatment: *p* = 0.5038; *F*_(1,64)_ = 0.45 Sex: *p* = 0.2468; *F*_(1,64)_ = 1.37 Genotype: *p* = 0.5155; *F*_(1,64)_ = 0.43 Treatment × sex: *p* = 0.3810; *F*_(1,64)_ = 0.78 Treatment × genotype: *p* = 0.0935; *F*_(1,64)_ = 2.90 Sex × genotype: *p* = 0.4062; *F*_(1,64)_ = 0.70 Treatment × sex × genotype: *p* = 0.7113; *F*_(1,64)_ = 0.14	Male WT SNI = 9 Male WT sham = 9 Male KO SNI = 10 Male KO sham = 10 Female WT SNI = 9 Female WT sham = 6 Female KO SNI = 9 Female KO sham = 10
7*H*	Time immobile in forced swim test at pre- and postsurgery	Three-way ANOVA with allowing different variances for genotype and adjusted for Bonferroni’s method (nonsignificant interaction effect dropped for post hoc purpose)	Treatment: *p = 0.0221; *F*_(1,66)_ = 5.50 Sex: *p* = 0.4848; *F*_(1,66)_ = 0.49 Genotype: *p* = 0.1756; *F*_(1,66)_ = 1.87 Sex × treatment: *p = 0.0440; *F*_(1,66)_ = 4.21 Genotype × treatment: *p = 0.0155; *F*_(1,66)_ = 6.17 Genotype × treatment effect: adjusted *p* value KO SNI vs KO sham: *p* = 0.9997 KO SNI vs WT SNI: *p = 0.0365 KO SNI vs WT sham: *p* = 0.9078 KO sham vs WT SNI: *p = 0.0421 KO sham vs WT sham: *p* = 0.8642 WT SNI vs WT sham: **p = 0.0021 Sex × treatment effect: adjusted *p* value Female SNI vs female sham: *p = 0.0186 Female SNI vs male SNI: *p* = 0.1972 Female SNI vs male sham: *p* = 0.1320 Female sham vs male SNI: *p* = 0.6580 Female sham vs Male sham: *p* = 0.7888 Male SNI vs male sham: *p* = 0.9953 Significant treatment post hoc effects SNI WT female vs sham WT female: ***p = 0.0003	Male WT SNI = 9 Male WT sham = 9 Male KO SNI = 10 Male KO sham = 10 Female WT SNI = 9 Female WT sham = 6 Female KO SNI = 9 Female KO sham = 10
7*I*	Time immobile in tail suspension test at pre- and postsurgery	Three-way ANOVA with allowing different variances for genotype and adjusted for Bonferroni’s method (nonsignificant interaction effect dropped for post hoc purpose)	Treatment: *p* = 0.6647; *F*_(1,65.3)_ = 0.19 Sex: *p = 0.0282; *F*_(1,60.6)_ = 5.06 Genotype: **p = 0.0066; *F*_(1,60.6)_ = 7.91 Genotype × sex: *p = 0.0321; *F*_(1,60.6)_ = 4.81 Genotype × sex effect: adjusted *p* value KO female vs KO male: *p* = 1.0000 KO female vs WT female: **p = 0.0060 KO female vs WT male: *p* = 0.9763 KO male vs WT female: **p = 0.0048 KO male vs WT male: *p* = 0.9685 WT female vs WT male: *p = 0.0323 Significant sex post hoc effects SNI WT male vs SNI WT female: *p* = 0.3273 Sham WT male vs sham WT female: *p* = 0.3798 Significant genotype post hoc effects SNI WT female vs SNI KO female: *p* = 0.5202 Sham WT female vs sham KO female: *p* = 0.0652	Male WT SNI = 9 Male WT sham = 9 Male KO SNI = 10 Male KO sham = 10 Female WT SNI = 9 Female WT sham = 6 Female KO SNI = 9 Female KO sham = 10
8	Mechanical hypersensitivity across time	Repeated-measures two-way ANOVA between time and group followed by Tukey's multiple-comparisons test at each time point	Time: ****p < 0.0001; *F*_(2.853,142.6)_ = 46.62 Group: ****p < 0.0001; *F*_(3,50)_ = 16.62 Time × group: ****p < 0.0001; *F*_(15,250)_ = 8.33 Baseline 1 WT SNI vs WT sham: *p* = 0.9594 WT SNI vs KO SNI: *p* = 0.9989 WT sham vs KO sham: *p* = 0.4629 KO SNI vs KO sham: *p* = 0.7256 Baseline 2 WT SNI vs WT sham: *p* = 0.9049 WT SNI vs KO SNI: *p* = 0.9661 WT sham vs KO sham: *p* = 0.2216 KO SNI vs KO sham: *p* = 0.9947 Post 7 d WT SNI vs WT sham: *p = 0.0418 WT SNI vs KO SNI: *p* = 0.9263 WT sham vs KO sham: *p* = 0.9921 KO SNI vs KO sham: **p = 0.0015 Post 14 d WT SNI vs WT sham: **p = 0.0022 WT SNI vs KO SNI: *p* = 0.7165 WT sham vs KO sham: *p* = 0.9520 KO SNI vs KO sham: ****p < 0.0001 Post 21 d WT SNI vs WT sham: ****p < 0.0001 WT SNI vs KO SNI: *p* > 0.9999 WT sham vs KO sham: *p* = 0.8188 KO SNI vs KO sham: ***p = 0.0003 Post 28 d WT SNI vs WT sham: ***p = 0.0002 WT SNI vs KO SNI: *p* = 0.9864 WT sham vs KO sham: *p* = 0.5849 KO SNI vs KO sham: ****p < 0.0001	WT sham = 11 WT SNI = 13 *Tmem97* KO sham = 16 *Tmem97* KO SNI = 14
3-1*A*	Elevated zero maze—total distance traveled by genotype	Two-way ANOVA with Tukey's multiple-comparisons test	Sex: *p* = 0.2066; *F*_(1,51)_ = 1.64 Genotype: *p* = 0.5897; *F*_(1,51)_ = 0.29 Interaction: *p* = 0.1637; *F*_(1,51)_ = 2.00	Male WT = 15 Female WT = 9 Male *Tmem97* KO = 16 Female *Tmem97* KO = 15
3-1*B*	Elevated zero maze—number of entries in open arm by genotype	Two-way ANOVA with Tukey's multiple-comparisons test	Sex: *p* = 0.2613; *F*_(1,51)_ = 1.29 Genotype: *p* = 0.6678; *F*_(1,51)_ = 0.19 Interaction: *p* = 0.5986; *F*_(1,51)_ = 0.28	Male WT = 15 Female WT = 9 Male *Tmem97* KO = 16 Female *Tmem97* KO = 15
3-1*C*	Elevated zero maze—time spent in open arm by genotype	Two-way ANOVA with Tukey's multiple-comparisons test	Sex: *p* = 0.1635; *F*_(1,51)_ = 2.00 Genotype: *p* = 0.5272; *F*_(1,51)_ = 0.41 Interaction: *p* = 0.5967; *F*_(1,51)_ = 0.28	Male WT = 15 Female WT = 9 Male *Tmem97* KO = 16 Female *Tmem97* KO = 15
8-1*A*	Time taken for walk initiation test	Repeated-measures mixed model ANOVA with allowing different variances for genotype and adjusted for Bonferroni’s method (nonsignificant interaction effect dropped for post hoc purpose)	Time (pre/post): *p* = 0.2076; *F*_(1,24)_ = 1.68 Genotype: *p* = 0.6009; *F*_(1,23)_ = 0.28 Treatment: *p* = 0.2600; *F*_(1,23)_ = 1.33 Time × treatment: *p = 0.0355; *F*_(1,24)_ = 4.96 Time × treatment effect: adjusted *p* value Pre SNI vs post SNI: *p* = 0.1041 Post sham vs post SNI: *p* = 0.2189 Pre sham vs post SNI: *p* = 0.3998 Pre SNI vs post sham: *p* = 0.9593 Pre sham vs pre SNI: *p* = 0.9996 Pre sham vs post sham: *p* = 0.9013	WT sham = 7 WT SNI = 8 *Tmem97* KO sham = 7 *Tmem97* KO SNI = 4
8-1*B*	Time taken to come down to the bottom of the pole	Repeated-measures mixed model ANOVA with allowing different variances for genotype	Time (pre/post): *p* = 0.1908; *F*_(1,22)_ = 1.82 Genotype: *p* = 0.8413; *F*_(1,22)_ = 0.04 Treatment: *p* = 0.8458; *F*_(1,22)_ = 0.04 Time × genotype: *p* = 0.8469; *F*_(1,22)_ = 0.04 Time × treatment: *p* = 0.1271; *F*_(1,22)_ = 2.51 Genotype × treatment: *p* = 0.3423; *F*_(1,22)_ = 0.94 Time × genotype × treatment: *p* = 0.2302; *F*_(1,22)_ = 1.52	WT sham = 7 WT SNI = 8 *Tmem97* KO sham = 7 *Tmem97* KO SNI = 4
8-1*C*	Time taken to make the first turn on the pole	Repeated-measures mixed model ANOVA with allowing different variances for genotype	Time (pre/post): *p* = 0.5788; *F*_(1,22)_ = 0.32 Genotype: *p* = 0.9752; *F*_(1,22)_ = 0.00 Treatment: *p* = 0.4152; *F*_(1,22)_ = 0.69 Time × genotype: *p* = 0.3913; *F*_(1,22)_ = 0.76 Time × treatment: *p* = 0.4790; *F*_(1,22)_ = 0.52 Genotype × treatment: *p* = 0.9425; *F*_(1,22)_ = 0.01 Time × genotype × treatment: *p* = 0.9324; *F*_(1,22)_ = 0.01	WT sham = 7 WT SNI = 8 *Tmem97* KO sham = 7 *Tmem97* KO SNI = 4
8-1*D*	Time stayed on the ledge without falling	Repeated-measures mixed model ANOVA with allowing different variances for genotype	Time (pre/post): *p* = 0.1869; *F*_(1,22)_ = 1.86 Genotype: *p* = 0.1869; *F*_(1,22)_ = 1.86 Treatment: *p* = 0.1869; *F*_(1,22)_ = 1.86 Time × genotype: *p* = 0.1869; *F*_(1,22)_ = 1.86 Time × treatment: *p* = 0.1869; *F*_(1,22)_ = 1.86 Genotype × treatment: *p* = 0.1869; *F*_(1,22)_ = 1.86 Time × genotype × treatment: *p* = 0.1869; *F*_(1,22)_ = 1.86	WT sham = 7 WT SNI = 8 *Tmem97* KO sham = 7 *Tmem97* KO SNI = 4
8-1*E*	Time stayed on the platform without falling	Repeated-measures Mixed Model ANOVA with allowing different variances for genotype	Time (pre/post): *p* = 0.3829; *F*_(1,22)_ = 0.79 Genotype: *p* = 0.3829; *F*_(1,22)_ = 0.79 Treatment: *p* = 0.3829; *F*_(1,22)_ = 0.79 Time × genotype: *p* = 0.3829; *F*_(1,22)_ = 0.79 Time × treatment: *p* = 0.3829; *F*_(1,22)_ = 0.79 Genotype × treatment: *p* = 0.3829; *F*_(1,22)_ = 0.79 Time × genotype × treatment: *p* = 0.3829; *F*_(1,22)_ = 0.79	WT sham = 7 WT SNI = 8 *Tmem97* KO sham = 7 *Tmem97* KO SNI = 4
8-1*F*	Latency time to go up to the top of the 60° inclined wire mesh	Repeated-measures mixed model ANOVA with allowing different variances for genotype	Time (pre/post): *p* = 0.4457; *F*_(1,22)_ = 0.60 Genotype: *p* = 0.2325; *F*_(1,22)_ = 1.51 Treatment: *p* = 0.6187; *F*_(1,22)_ = 0.25 Time × genotype: *p* = 0.8701; *F*_(1,22)_ = 0.027 Time × treatment: *p* = 0.5581; *F*_(1,22)_ = 0.35 Genotype × treatment: *p* = 0.4541; *F*_(1,22)_ = 0.58 Time × genotype × treatment: *p* = 0.3706; *F*_(1,22)_ = 0.84	WT sham = 7 WT SNI = 8 *Tmem97* KO sham = 7 *Tmem97* KO SNI = 4
8-1*G*	Latency time to go down to the bottom of the 60° inclined wire mesh	Repeated-measures mixed model ANOVA with allowing different variances for genotype	Time (pre/post): ****p < 0.0001; *F*_(1,22)_ = 33.51 Genotype: *p* = 0.1658; *F*_(1,22)_ = 2.05 Treatment: *p* = 0.8178; *F*_(1,22)_ = 0.05 Time × genotype: *p* = 0.1658; *F*_(1,22)_ = 2.05 Time × treatment: *p* = 0.8178; *F*_(1,22)_ = 0.05 Genotype × treatment: *p* = 0.7309; *F*_(1,22)_ = 0.12 Time × genotype × treatment: *p* = 0.7309; *F*_(1,22)_ = 0.12	WT sham = 7 WT SNI = 8 *Tmem97* KO sham = 7 *Tmem97* KO SNI = 4
8-1*H*	Latency time to go up to the top of the 90° inclined wire mesh	Repeated-measures mixed model ANOVA with allowing different variances for genotype	Time (pre/post): ***p = 0.0006; *F*_(1,22)_ = 15.81 Genotype: *p* = 0.3877; *F*_(1,22)_ = 0.78 Treatment: *p* = 0.4373; *F*_(1,22)_ = 0.63 Time × genotype: *p* = 0.2603; *F*_(1,22)_ = 1.34 Time × treatment: *p* = 0.1912; *F*_(1,22)_ = 1.82 Genotype × treatment: *p* = 0.2726; *F*_(1,22)_ = 1.27 Time × genotype × treatment: *p* = 0.4752; *F*_(1,22)_ = 0.53	WT sham = 7 WT SNI = 8 *Tmem97* KO sham = 7 *Tmem97* KO SNI = 4
8-1*I*	Latency time to go down to the bottom of the 90° inclined wire mesh	Repeated-measures mixed model ANOVA with allowing different variances for genotype	Time (pre/post): **p = 0.0053; *F*_(1,22)_ = 9.59 Genotype: *p* = 0.0704; *F*_(1,22)_ = 3.62 Treatment: *p* = 0.4517; *F*_(1,22)_ = 0.59 Time × genotype: *p* = 0.0704; *F*_(1,22)_ = 3.62 Time × treatment: *p* = 0.4517; *F*_(1,22)_ =0.59 Genotype × treatment: *p* = 0.6716; *F*_(1,22)_ = 0.18 Time × genotype × treatment: *p* = 0.6716; *F*_(1,22)_ = 0.18	WT sham = 7 WT SNI = 8 *Tmem97* KO sham = 7 *Tmem97* KO SNI = 4
8-1*J*	Latency time to fall from inversed wire mesh	Repeated-measures mixed model ANOVA with allowing different variances for genotype	Time (pre/post): **p = 0.0086; *F*_(1,22)_ = 8.32 Genotype: *p* = 0.6758; *F*_(1,22)_ = 0.18 Treatment: *p* = 0.2138; *F*_(1,22)_ = 1.64 Time × genotype: *p* = 0.3852; *F*_(1,22)_ = 0.78 Time × treatment: *p* = 0.5172; *F*_(1,22)_ = 0.43 Genotype × treatment: *p* = 0.9073; *F*_(1,22)_ = 0.01 Time × genotype × treatment: *p* = 0.5435; *F*_(1,22)_ = 0.38	WT sham = 7 WT SNI = 8 *Tmem97* KO sham = 7 *Tmem97* KO SNI = 4

Statistical significance was determined at the level of *p* < 0.05. Asterisks denoting *p* values include *p < 0.05, **p < 0.01, ***p < 0.001, and ****p < 0.0001. All data are presented as the mean ± standard error of the mean (SEM).

#### Z score calculation

*Z* score analysis demonstrates how many standard deviations (*σ*) an observation (*X*) is above or below the mean of a control group (*μ*) to determine overall behavioral phenotypes (Guilloux et al., 2011). To calculate the overall *Z* score for an individual assay, individual parameters were selected and normalized using the following formula (Guilloux et al., 2011):

$$Z_{\mathrm{OF}}=\frac{\left(\frac{X-\mu }{\sigma }\right)\mathrm{TO}+\;\left(\frac{X-\mu }{\sigma }\right)\mathrm{PO}}{\mathrm{Number}\;\mathrm{of}\;\mathrm{parameters}}.$$

For example, the overall *Z* score for open field was calculated by summing the individual mouse *Z* score for time spent in the outside zone (TO) with the corresponding *Z* score for percent distance in outside zone (PO). For the other assays, various parameters were used that did not mathematically cancel each other out. For light/dark preference, four parameters were used: time spent in the light side, number of entries to the light side, latency to enter the light side, and mean time spent in the light side per visit. For elevated plus maze and elevated zero maze, two parameters were used: time spent in open arm and percent distance in open arm. For forced swim test and tail suspension test, the only parameter that was measured, and therefore used, was the time spent immobile. These *Z* scores were calculated within each assay, we next calculated the “emotionality” *Z* score to provide an overall picture of an affective behavioral phenotype. This was calculated by averaging all the *Z* scores from each individual assay, as shown below (Lax et al., 2018):

$$\mathrm{Emotionality}\;\mathrm{Score}=\frac{Z_{\mathrm{OF}}+Z_{\mathrm{LDP}}+Z_{\mathrm{EPM}}+Z_{\mathrm{EZM}}+Z_{\mathrm{FST}}+Z_{\mathrm{TST}}}{\mathrm{Number}\;\mathrm{of}\;\mathrm{assays}}.$$

The directionality of anxiety (or depressive) state in each parameter was controlled by the sign of values, with more positive values indicating increased anxiety or depressive-like behaviors.

## Results

### Evaluation of *Tmem97* expression in global *Tmem97* knock-out and wild-type mice

**Figure 1.**
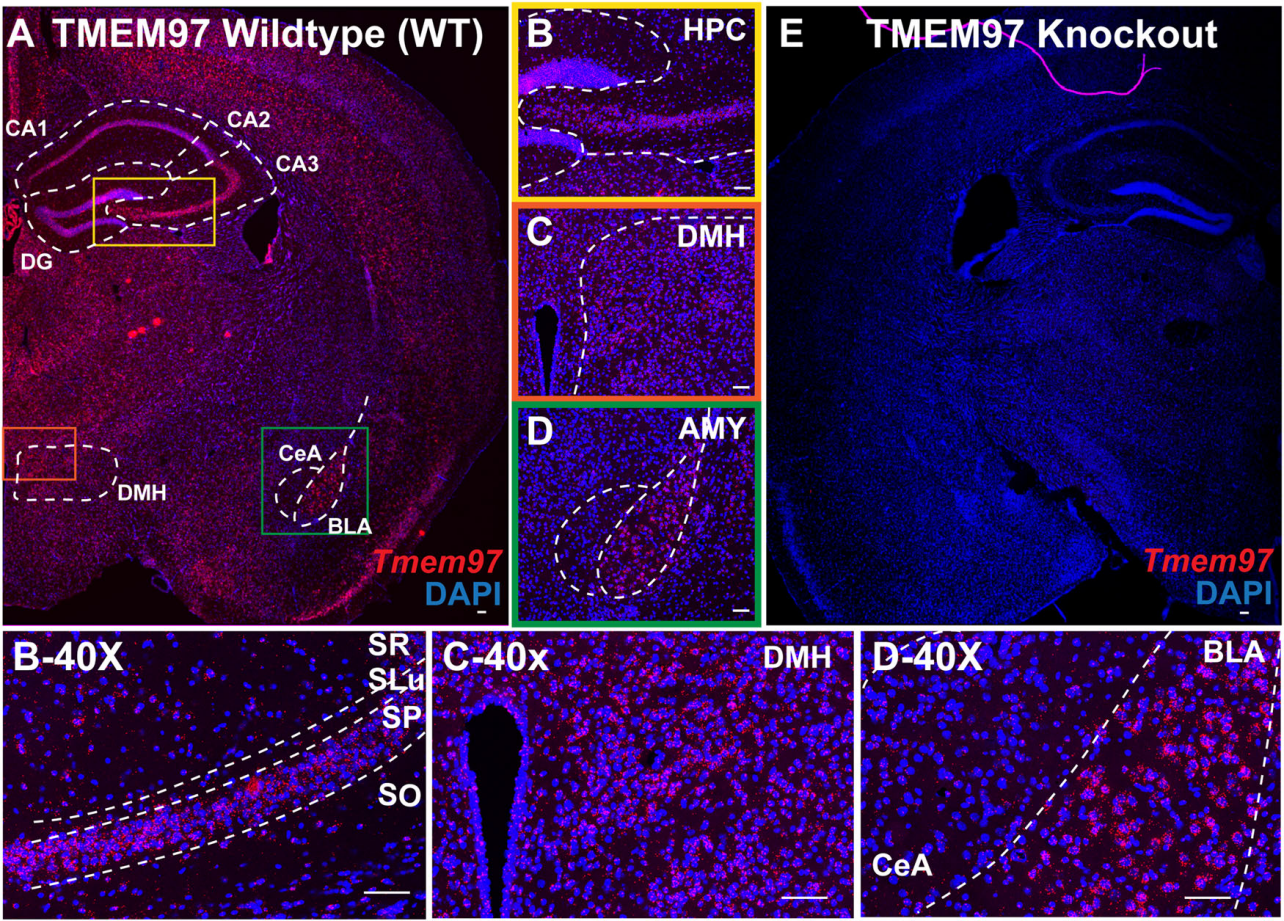
Visualization of *Tmem97* expression in wild-type and global *Tmem97* KO mice. Representative brain images of (***A***) WT and (***E***) global *Tmem97* KO mice are labeled with in situ hybridization for *Tmem97* (red) with DAPI (blue). Representative images for different regions of WT are shown including the (***B***) hippocampus, (***C***) dorsomedial hypothalamus, and (***D***) amygdala. Corresponding regions imaged at 40× with zoomed field of view are shown in the bottom panel: (***B-40X***) CA3 hippocampal layers, (***C-40x***) DMH, and (***D-40X***) amygdala subregions. DG, dentate gyrus; CeA, central amygdala; BLA, basolateral amygdala; DMH, dorsomedial hypothalamus; SR, stratum radiatum; SLu, stratum lucidum; SP, stratum pyramidal; SO, stratum oriens. Scale bar, 100 μm.

We first validated whether the expression *Tmem97* was absent in the global knock-out (KO) mice compared with wild-type (WT) mice. WT *Tmem97* expression (Fig. 1*A*) was absent in *Tmem97* KO mice (Fig. 1*E*) as evaluated with in situ hybridization. In WT mice, *Tmem97* was expressed over most brain regions with varying levels (Fig. 1*A*). We observed *Tmem97* expression in brain structures of interest, like the hippocampus (Fig. 1*B*), dorsomedial hypothalamus (DMH; Fig. 1*C*), and amygdala (Fig. 1*D*). In hippocampal CA3 layers, *Tmem97* was expressed relatively higher in stratum pyramidal layers compared with other layers (Fig. 1*B-40X*). We also observed *Tmem97* expression in the DMH that are sporadically spread out in the region (Fig. 1*C-40x*). In the amygdala, *Tmem97* expression localized more to the basolateral amygdala (BLA) compared with central amygdala (CeA; Fig. 1*D-40X*). Here, we have validated the absence of *Tmem97* expression in the *Tmem97* KO mice and shown differential distribution of *Tmem97* expression in brain regions of WT mice.

### The loss of *Tmem97* is associated with reduced anxiety-like and depressive-like behaviors in light/dark preference and tail suspension test

**Figure 2.**
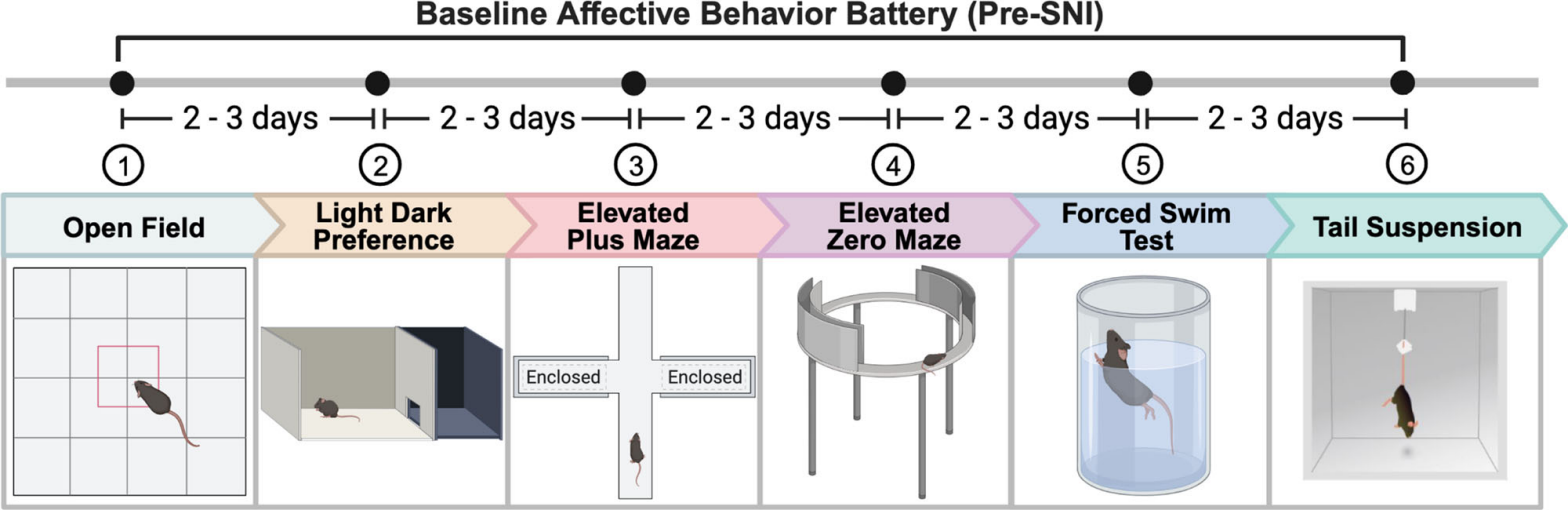
Experimental timeline on baseline affective behaviors. Affective behaviors are performed on both WT and *Tmem97* KO mice. Affective behaviors are performed in the order of open field, light/dark preference, elevated plus maze, elevated zero maze, forced swim test, and tail suspension test with 2 or 3 d in between the tests.

To test the behavioral consequence of s_2_R/TMEM97 loss in modulating affective behaviors in the naive state, we evaluated anxiety-like and depressive-like behaviors using an affective behavior battery in WT and *Tmem97* KO mice. Our primary battery included open field (OF), light and dark preference (LDP), elevated plus maze (EPM), elevated zero maze (EZM), forced swim test (FST), and tail suspension test (TST). We tested this affective behavioral battery (Fig. 2) on WT and *Tmem97* KO mice of both sexes with 2–3 d in between each behavioral assay.

**Figure 3.**
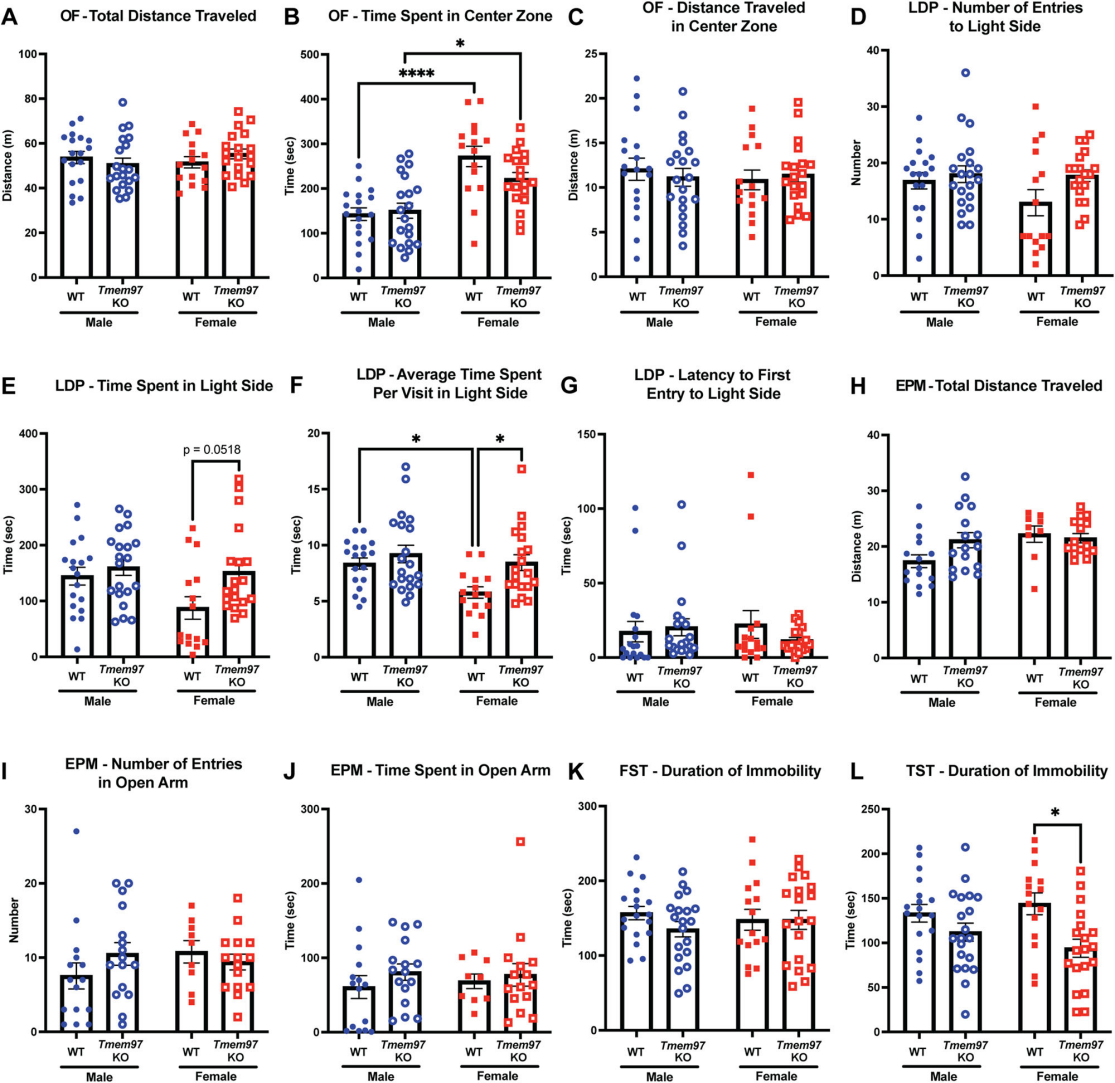
Affective behaviors between wild-type and global *Tmem97* KO naive mice. In naive mice, (***A–C***) there are no statistically significant differences in open-field behavior between WT and KO mice. ***B***, Female mice show less anxiety compared with male mice irrespective of genotype. ***D–F***, In the LDP test, female WT mice tend to show increased anxiety-like behaviors compared with female *Tmem97* KO mice with a statistically significant difference observed in the (***F***) average time spent per visit. ***G***, There was no trend for a difference in the LDP for latency to enter the light zone. ***H–J***, In the EPM, there are no significant differences between WT and KO mice nor any obvious sex differences. Extended Data Figure 3-1 includes anxiety-like behaviors between WT and *Tmem97* KO mice in both sexes using the EZM, another anxiety-like behavioral assay similar to EPM. ***K***, In the FST, there are no differences between genotypes and sex in immobility behavior. ***L***, In the TST, female *Tmem97* KO mice show less depressive-like behaviors than female WT mice. OF, open field; LDP, light/dark preference; EPM, elevated plus maze; EZM, elevated zero maze; FST, forced swim test; TST, tail suspension test. Two-way ANOVA with Tukey's multiple comparison. Values are mean ± SEM, significant difference *p < 0.05, ****p < 0.0001.

Our findings indicated that *Tmem97* KO female mice show reduced anxiety-like behaviors in LDP and TST but not in OF, EPM, EZM, and FST. The data are presented here in the order that mice were tested. First, no significant genotype difference is observed between WT and *Tmem97* KO mice in either sex with the following OF parameters: total distance traveled (Fig. 3*A*), time spent in center zone (Fig. 3*B*), and distance traveled in center zone (Fig. 3*C*). One of the OF parameters, time spent in center zone, showed a statistically significant main sex effect (*F*_(1,68)_ = 35.04; ****p < 0.0001) with Tukey's post hoc analysis showing both significant differences in WT (male WT vs female WT, ****p < 0.0001) and *Tmem97* KO mice (male KO vs female KO, *p = 0.0160; Fig. 3*B*). Females, both WT and *Tmem97* KO, spent more time in the center zone compared with male mice. Female mice displayed less anxiety-like behaviors that are not associated with genotype (Fig. 3*B*). There was no sex difference within the same genotype for the total distance traveled or the distance traveled in center zone (Fig. 3*A*,*C*).

10.1523/ENEURO.0488-23.2024.f3-1 Figure 3-1 **No significant difference between anxiety-like behaviors between wild-type and *Tmem97* KO mice in both sexes using elevated zero maze.** **(*A, B, C*)** In naïve mice, no significant difference between genotypes in both sexes is observed in any parameter of the EZM: total distance traveled, number of entries in open arm, and time spent in open arm. EZM = elevated zero maze. [Two-way ANOVA with Tukey's multiple comparison test]. Values are mean +/- SEM. Download Figure 3-1, TIF file.

In LDP, we found that female *Tmem97* KO mice show a trend of reduced anxiety-like behaviors in certain parameters compared with female WT mice. Compared with female WT mice, female *Tmem97* KO mice showed a trend for increased number of visits (Fig. 3*D*) and time spent (Fig. 3*E*) in the light chamber with a statistically significant increase in the average time spent per visit to the light chamber (Fig. 3*F*). For the time spent in light side parameter, we observed a statistically significant main effect of genotype (*F*_(1,68)_ = 5.63; *p = 0.0205; Fig. 3*E*). For average time spent per visit in light side, we observed a main sex effect (*F*_(1,68)_ = 6.40; *p = 0.0137) and main genotype effect (*F*_(1,68)_ = 7.08; **p = 0.0097) but not a significant interaction effect (*F*_(1,68)_ = 1.88; *p* = 0.1752). With Tukey's post hoc analysis, the sex effect was seen only within WT mice (male WT vs female WT, *p = 0.0487), and the genotype effect was seen only within female mice (female WT vs female KO, *p = 0.0358; Fig. 3*F*). The observed difference in genotypes among female mice appears to be due to an increase in anxiety-like behaviors that is specific to females when compared with their male WT controls. No effect of genotype was observed in latency to enter the light chamber (Fig. 3*G*). The overall result reflects that *Tmem97* KO mice show higher exploratory activity in the novel environment despite their innate tendency to favor the dark protected chamber as shown in other measurements of the LDP assay. The genotypic difference observed in LDP measurements may be driven by a female-specific interaction with the s_2_R/TMEM97.

The EPM and EZM are two behavioral assays that measure time in a protected area (closed arms) and an unprotected area (open arms; Walf and Frye, 2007). No significant difference was observed in the total distance traveled in either EPM or EZM between genotypes (Fig. 3*H–J*; Extended Data Fig. 3-1*A–C*). For the total distance traveled in EPM, no significant difference was observed except for the main effect of sex (*F*_(1,51)_ = 4.35; *p = 0.0421; Fig. 3*H*). This reflects that the effect of genotype observed above in LDP is not likely due to locomotor activity in female mice.

The last two assays of the affective behavioral battery were the FST and the TST. For the FST, we did not observe any statistical significance in the main effect or the interaction effect. Both WT and *Tmem97* KO mice showed similar levels of immobility behavior irrespective of sex (Fig. 3*K*). For TST, we observed a main effect of genotype (*F*_(1,68)_ = 11.24; *p = 0.0013) but not a main effect of sex (*F*_(1,68)_ = 0.12; *p* = 0.7254), nor an interaction effect (*F*_(1,68)_ = 1.84; *p* = 0.1799). With a post hoc analysis, a genotype effect was seen only within female mice (female WT vs female KO, *p = 0.0100) but not in male mice (male WT vs male KO, *p* = 0.4683). The female *Tmem97* KO mice show a statistically significant reduction in depressive-like behaviors, reflected by shorter duration of immobility in TST (Fig. 3*L*). Here, the behavioral consequences of *Tmem97* loss in affective behaviors showed significant changes in anxiety-like and depressive-like behaviors across various tests in female mice, but these effects appear to be test specific.

### The loss of *Tmem97* is associated with a decrease in overall “emotionality,” demonstrated with an integrated behavioral *Z* score

Taken together, the loss of *Tmem97* expression is associated with reduced anxiety-like and depressive-like behaviors in LDP and TST, respectively. However, these *Tmem97*-associated affective behavior phenotypes appear to only be associated with a specific set of tests but not necessarily in similar tests. For instance, there is a lack of consistency between FST and TST results, even though both assays are commonly described in terms of testing for “depressive-like” states. Such discrepancies were also observed in anxiety tests like OF, LDP, EPM, and EZM. This may be due to an intrinsic variability of a single test (Palanza, 2001; Võikar et al., 2001; Carola et al., 2002; Ramos, 2008) or an underlying mechanism of affective behavior in rodents (Dalla et al., 2005; Joeyen-Waldorf et al., 2009), given that certain antidepressants are effective exclusively in particular models of depressive states (Kulkarni and Dhir, 2007; Chatterjee et al., 2012). To better characterize *Tmem97*-associated emotionality in rodents, we used a *Z* score normalization (Guilloux et al., 2011).

Relative behavioral parameters were chosen to calculate *Z* scores within each test to prevent the confounding effect of measuring the same result using different approaches. For OF, we have selected time spent in outside zone and percent distance in outside zone. For LDP, all measured parameters were used, including number of entries to light side, time spent in light side, latency to light side, and mean visit to light side. For EPM and EZM, time spent in open arm and number of entries to the open arms were used. For FST and TST, only immobility time was used. WT male mice were used as the reference group.

**Figure 4.**
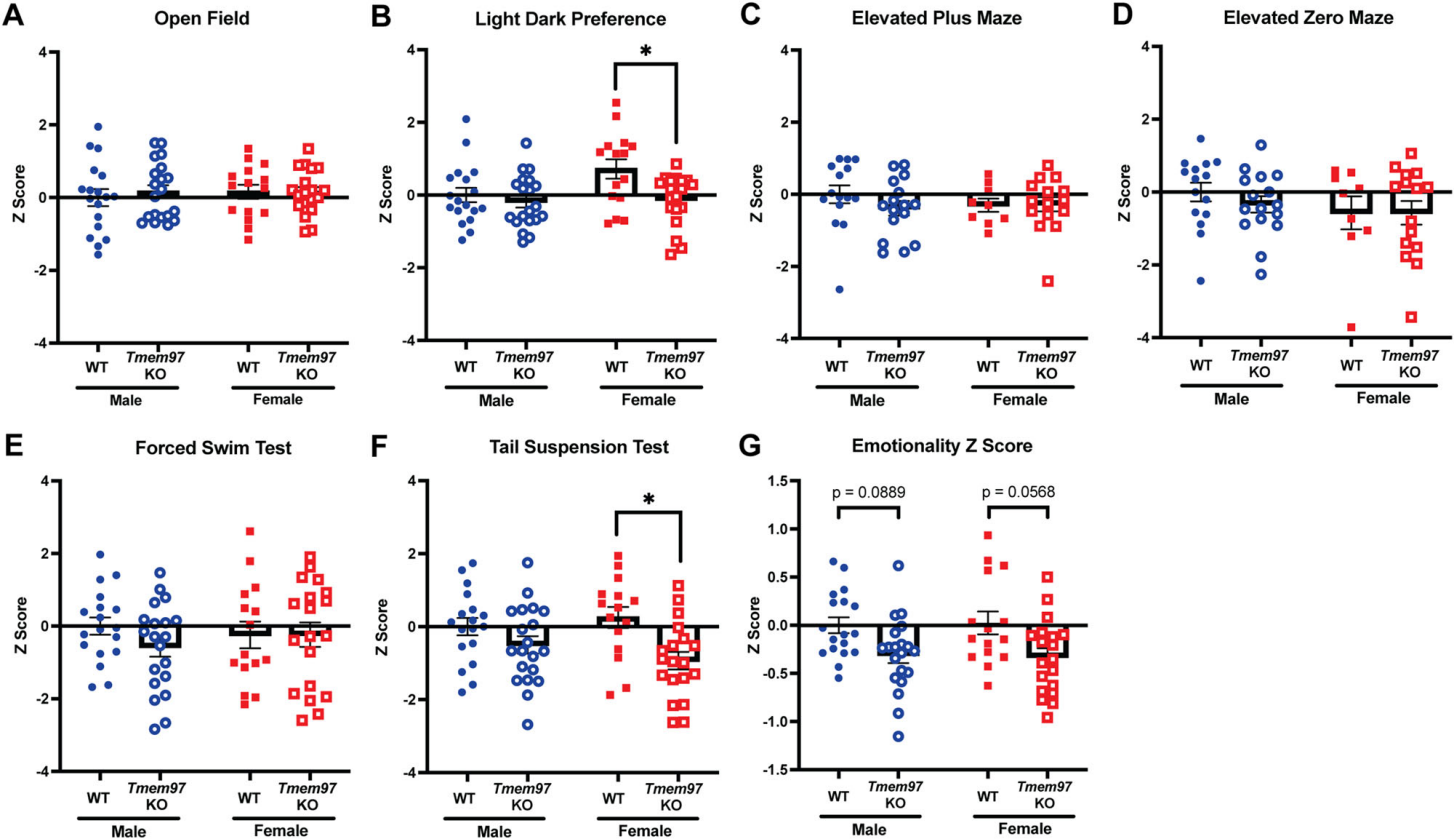
Analysis of test-specific and overall emotionality *Z* score to show combined effect of anxiety-like and depressive-like behaviors on wild-type and *Tmem97* KO mice. The effect of combined anxiety-like and depressive-like behaviors across different parameters for each behavioral assay was calculated using *Z* score normalization. Lower value in *Z* score indicates less anxiety-like and depressive-like phenotypes. No significant difference was observed between WT and *Tmem97* KO in *Z* score for (***A***) OF, (***C***) EPM, (***D***) EZM, and (***E***) FST. However, (***B***) in the LDP assay, female *Tmem97* KO mice displayed a significant less *Z* score than wild-type mice. ***F***, In the TST assay, female *Tmem97* KO mice displayed a significantly less *Z* score than wild-type mice. ***G***, The overall emotionality score shows that *Tmem97* KO mice show less combine anxiety-like and depressive-like behavior compared with WT mice (main effect of genotype *p* = 0.0008). OF, open field; LDP, light/dark preference; EPM, elevated plus maze; EZM, elevated zero maze; FST, forced swim test; TST, tail suspension test. Two-way ANOVA with Tukey's multiple comparison. Values are mean ± SEM, significant difference *p < 0.05.

For the test-specific analysis, we found that female *Tmem97* KO mice had a significantly more negative *Z* score (i.e., antianxiety and antidepressant like) in the LDP (Fig. 4*B*) and the TST (Fig. 4*F*) but not in other affective tests (Fig. 4*A*,*C–E*). In LDP, we observed a main effect of genotype (*F*_(1,68)_ = 6.90; *p = 0.0106). Post hoc analysis with Tukey's showed a statistically significant difference within female mice (female WT vs female KO, *p = 0.0210). We found that WT female mice have an elevated *Z* score that is associated with high anxiety-like behavior compared with WT males, *Tmem97* KO males, and *Tmem97* KO female mice (Fig. 4*B*). Thus, the loss of *Tmem97* associated with less anxiety-like behaviors appears to be driven by WT female mice in LDP (Fig. 4*B*). Also, we found TST behavior showed a sex dimorphism underlying the loss of *Tmem97* associated with less depressive-like behaviors. We observed a main effect of genotype (*F*_(1,68)_ = 11.24; **p = 0.0013). Post hoc analysis showed a statistically significant difference within female mice (female WT vs female KO, *p = 0.0100). Female *Tmem97* KO mice had a significantly higher negative score associated with a less depressive-like state in TST (Fig. 4*F*). Both male and female *Tmem97* KO mice showed a decrease in *Z* score associated with less depressive-like behaviors compared with WT mice (Fig. 4*G*).

We then averaged the normalized *Z* scores for each behavior tests to obtain a single “emotionality” score to evaluate the effect of genotype (Guilloux et al., 2011; Lax et al., 2018). In the single overall “emotionally” *Z* score, we observed a statistically significant main genotype effect (*F*_(1,68)_ = 12.37; ***p = 0.0008) between WT and *Tmem97* KO mice (Fig. 4*G*). Post hoc analysis of the “emotionality” *Z* score by sex shows a similar trend of reduced anxiety- and depressive-like behaviors associated with the loss of *Tmem97* in both females (*p* = 0.0568) and males (*p* = 0.0889; Fig. 4*G*). This demonstrates that the loss of *Tmem97* is associated with a modest reduction in affective behavior. Overall, *Z* score on the effect of sex and genotype across behavioral dimensions provides a supporting assessment of how the loss of *Tmem97* leads to changes in affective behavioral outcomes.

### Loss of *Tmem97* is not associated with disruption in executive function and learning

**Figure 5.**
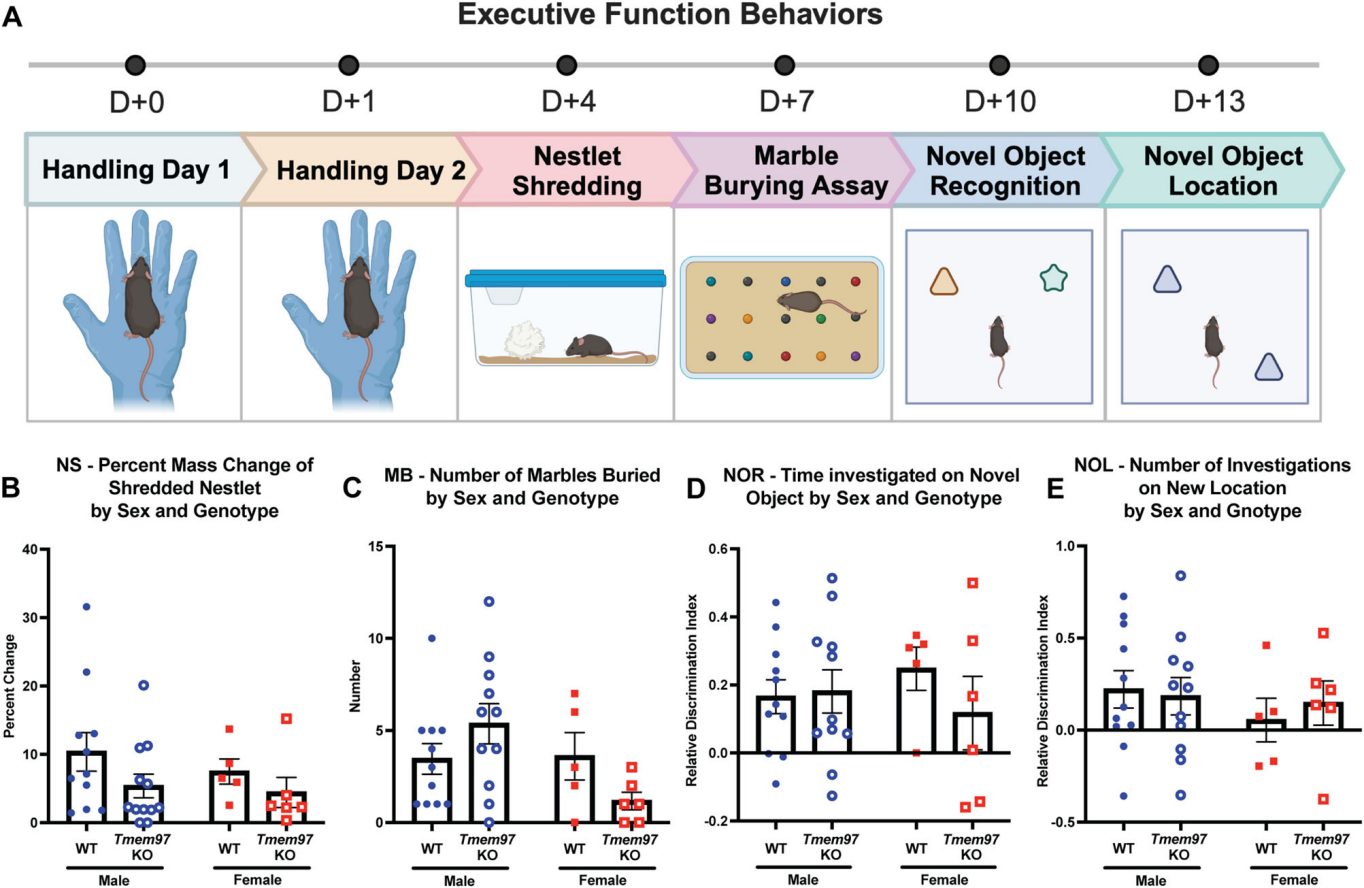
*Tmem97* is not associated with other behaviors related to executive function. ***A***, Experimental timeline on behaviors testing executive functions is as follows. After handling for 2 consecutive days, behaviors consisting of nestlet shredding, marble burying assay, novel object recognition, and novel object location are performed on both WT and *Tmem97* KO mice. No effect of sex or genotype was observed in (***B***) nestlet shredding, (***C***) marble burying, (***D***) novel object recognition, or (***E***) novel object location. NS, nestlet shredding, MB, marble burying, NOR, novel object recognition; NOL, novel object location. Relative discrimination index = [novel object (location) − familiar object (location)] / total exploration time of objects (location) (two-way ANOVA with Tukey's multiple-comparisons test). Values are mean ± SEM.

Finally, in naive animals, we tested the impact of *Tmem97* disruption on tasks associated with executive function and learning. We used marble burying and nestlet shredding test to investigate the compulsive, repetitive behaviors that are often observed in neurodevelopmental disorders involving deficits in sensory processing (Boyd et al., 2009). We also performed novel object recognition and novel object location to test the memory performance on object and spatial discrimination. Every cohort of mice went through 2 consecutive days of handling followed by a series of the four behaviors: nestlet shredding, marble burying, novel object recognition, and novel object location (Fig. 5*A*). We stratified the genotypes by sex to see whether there is a female-specific *Tmem97-*associated difference as observed above. We did not see any effect of genotype within sex across different behaviors testing executive functions (Fig. 5*B–E*).

### Prolonged exposure to neuropathic injury leads to *Tmem97*-associated depressive-like behaviors in female mice

**Figure 6.**
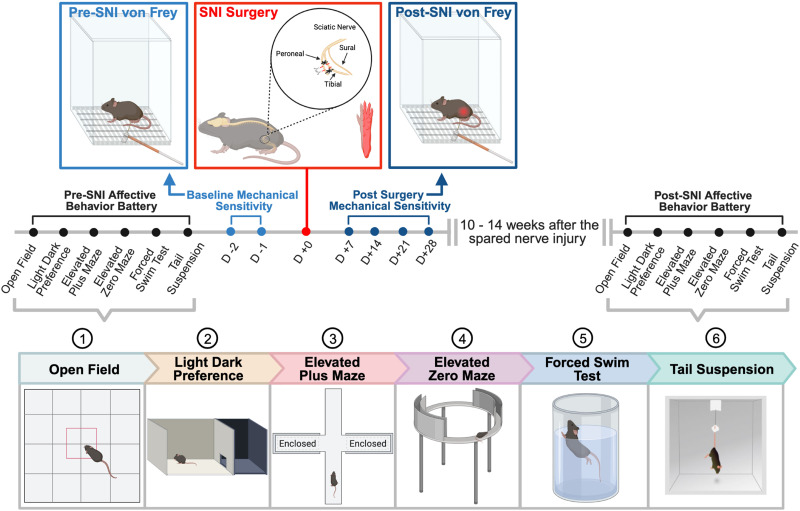
Experimental timeline on prolonged neuropathic pain-induced affective behaviors. Affective behaviors (open field, light/dark preference, elevated plus maze, elevated zero maze, forced swim test, and tail suspension test) were performed on both WT and *Tmem97* KO mice. Schematic of the SNI shows that the tibial and peroneal nerve are ligated and cut, leaving the sural nerve intact. Mechanical sensitivity is measured 2 d before the SNI and once every week (for 4 weeks) after the SNI was performed on a side of paw (in red) ipsilateral to injury. The affective behaviors are repeated >10 weeks after the SNI in the same manner as before the injury.

We next investigated the extent to which *Tmem97* is associated with the development of anxiety-like and depressive-like behaviors induced by prolonged neuropathic injury. After >10 weeks of neuropathic injury, we repeated the affective behavior battery (Fig. 6) described above. We stratified the postoperative behavioral changes by sex to investigate the potential sexual dimorphism underlying *Tmem97*-associated anxiety-like or depressive-like behaviors. We calculated the absolute change for mice by determining the numerical difference of the measured parameters of the assay from pre-SNI (or pre-sham) to post-SNI (or post-sham) for each mouse.

**Figure 7.**
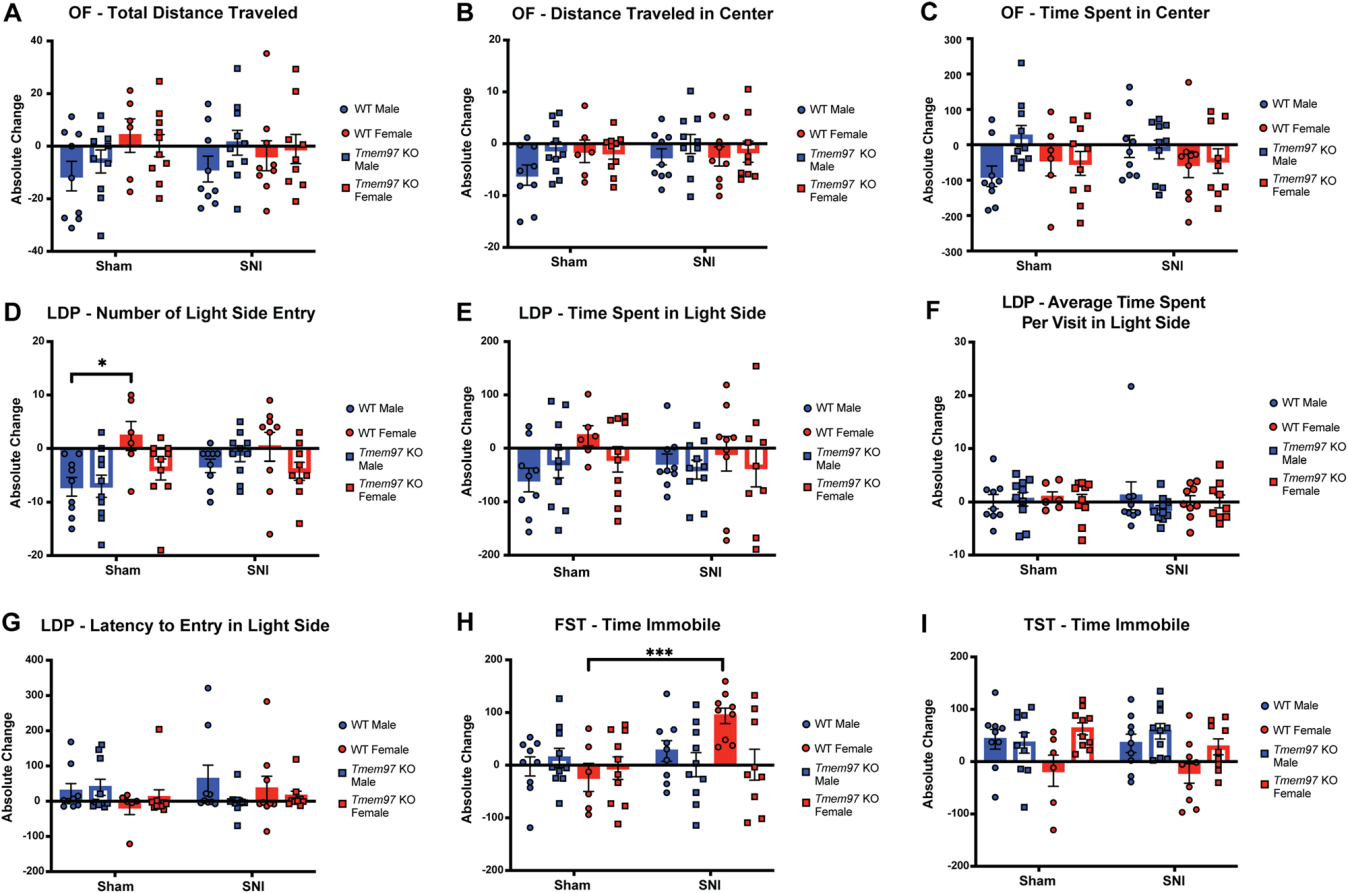
Postoperative absolute change in affective behaviors from baseline to after the surgery stratified by sex and genotype. ***A–C***, In OF, there were no significant differences between groups for absolute change from baseline. ***D–G***, In LDP, there was a sex difference in absolute change from baseline within WT sham mice for (***D***) the number of light entries only. ***H***, In the FST, female WT with SNI show significantly higher absolute changes from baseline compared with female sham WT mice consistent with a SNI-induced depressive-like effect in WT mice. ***I***, In the TST, there is no statistically significant difference among groups. OF, open field; LDP, light/dark preference; FST, forced swim test; TST, tail suspension test. Three-way ANOVA allowing for different variances for genotype and adjusted for Bonferroni’s post hoc analysis. Values are mean ± SEM, significant difference *p < 0.05 and ***p < 0.001.

In the OF test, we found no significant difference between WT and *Tmem97* KO mice after surgery, and no significant sex difference associated with genotype in the following parameters: total distance traveled (Fig. 7*A*), distance traveled in center (Fig. 7*B*), and time spent in center (Fig. 7*C*). In the LDP test, however, we observed a statistically significant main effect of sex (*F*_(1,62)_ = 5.40; *p = 0.0235) and significant interaction effects of sex × treatment (*F*_(1,65.4)_ = 4.92; *p = 0.0300) and genotype × sex (*F*_(1,62.1)_ = 5.46; *p = 0.0227) on the number of light side entries (Fig. 7*D*). *Post hoc* Bonferroni’s analysis of sex found a statistically significant difference within the sham group (male sham vs female sham, *p = 0.0128) and within the WT mice overall (male WT vs female WT, *p = 0.0177). When evaluating *post hoc* effect for both treatment and genotype, male WT sham mice showed a statistically significant difference compared with female WT sham mice (*p = 0.0011) on the number of light side entries. In other parameters measured in LDP, we failed to see any statistically significant differences among groups (Fig. 7*E–G*).

Interestingly, FST showed a statistically significant main effect of treatment (*F*_(1,66)_ = 5.50; *p = 0.0221) and significant interaction effects of sex × treatment (*F*_(1,66)_ = 4.21; *p = 0.0440) and genotype × treatment (*F*_(1,66)_ = 6.17; *p = 0.0155; Fig. 7*H*). Genotype × treatment Bonferroni’s *post hoc* effects were observed within the WT group (WT SNI vs WT sham, **p = 0.0021) and were not observed in the KO group (KO SNI vs KO sham, *p* = 0.9997). Sex × treatment *post hoc* effects were observed in female mice (female SNI vs female sham, *p = 0.0186) but not in male mice (male SNI vs male sham, *p* = 0.9953). *Post hoc* analysis of sex effects found statistically significant effects only in the WT sham group (WT sham male vs WT sham female, *p = 0.0011) but not in WT SNI group (WT SNI male vs WT SNI female, *p* = 0.3324). Altogether, the results show the presence of an SNI-induced depressive-like phenotype in female WT mice and that the loss of *Tmem97* protects mice from this outcome.

In the TST, we found a statistically significant main effect of sex (*F*_(1,60.6)_ = 5.06; *p = 0.0282), main effect of genotype (*F*_(1,60.6)_ = 7.91; **p = 0.0066), and significant interaction effect of genotype × sex (*F*_(1,60.6)_ = 4.81; *p = 0.0321; Fig. 7*I*). Bonferroni’s *post hoc* analysis did not find statistically significant differences between paired comparisons. In the TST in both sham and SNI groups, we found no *post hoc* significant differences between male and female WT mice (WT male sham vs WT female sham, *p* = 0.3798; WT male SNI vs WT female SNI, *p* = 0.3273). In both sham and SNI groups, there were trends for differences between WT and KO with the female sham group (WT sham female vs KO sham female, *p* = 0.0652). The SNI-induced depressive-like behavior observed in the FST test above is not observed in TST test, suggesting an assay-dependent *Tmem97*-associated pain-induced affective behavior phenotype.

### The antidepressant-like activity in *Tmem97* KO mice is independent of the pain-like mechanical hypersensitivity caused by neuropathic injury

**Figure 8.**
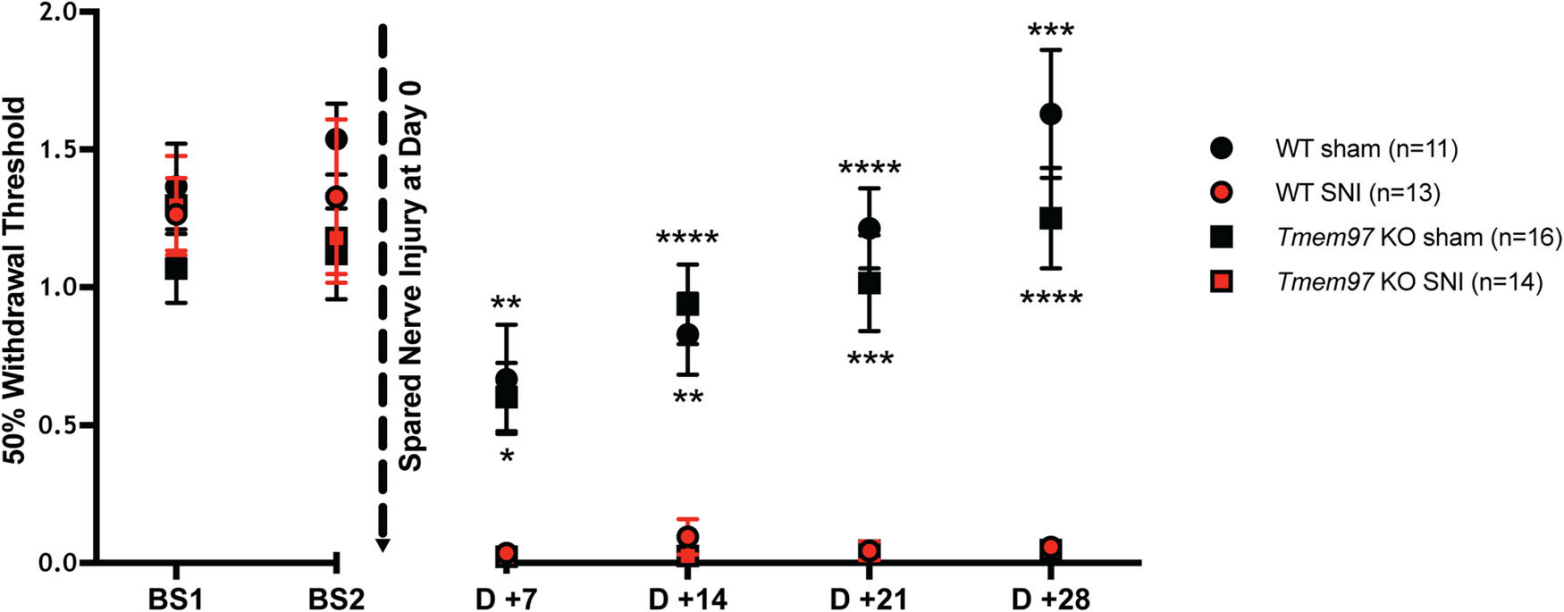
Mechanical hypersensitivity before and after the SNI in wild-type and *Tmem97* KO mice. No significant difference of mechanical hypersensitivity between WT and *Tmem97* KO mice was observed at baseline and after SNI or sham surgery. Prolonged mechanical hypersensitivity was induced by SNI for WT and *Tmem97* KO mice. Sham-treated group gradually recovered from acute mechanical hypersensitivity with full recovery by 4 weeks. Sham groups show statistically significant differences from the SNI group regardless of genotype. WT SNI (*n* = 13, 7 male and 6 female). WT sham (*n* = 11, 8 male and 3 female). *Tmem97* KO SNI (*n* = 14, 7 male and 7 female). *Tmem97* KO sham (*n* = 16, 8 male and 8 female). Extended Data Figure 8-1 shows a series of sensory-motor behavioral batteries performed with WT and *Tmem97* KO mice to demonstrate that no motor deficits are observed, and affective behavioral phenotypes are independent of the surgery. BS, baseline. Repeated-measures, two-way ANOVA between group and time with Tukey's multiple-comparisons test within each time point. Values are mean ± SEM, no significant difference between the genotype within the same treatment. Significant difference between the treatment within the same genotype *p < 0.05, **p < 0.01, ***p < 0.001, ****p < 0.0001.

We evaluated the impact of SNI neuropathic injury on peripheral mechanical sensitivity in a subset of mice. This was to ensure that the observed antidepressant-like effects in *Tmem97* KO mice were not due to differences in their susceptibility to developing pain-like mechanical hypersensitivity, which could be influenced by *Tmem97*. The mechanical sensitivity testing showed a statistically significant main effect of time (*F*_(2.853,142.6)_ = 46.62; ****p < 0.0001), main effect of groups (*F*_(3,50)_ = 16.62; ****p < 0.0001), and an interaction effect (*F*_(15,250)_ = 8.33; ****p < 0.0001; Fig. 8). At baseline (prior to surgery), *post hoc* analysis found no significant difference in mechanical sensitivity between the genotypes. After SNI (or control sham) surgeries, mechanical sensitivity was measured every week for 4 weeks. At each time point, both WT and *Tmem97* KO mice with SNI showed increased mechanical sensitivity compared with WT and *Tmem97* KO mice with control sham surgeries at 7 d postsurgery (WT SNI vs WT sham, *p = 0.0418; KO SNI vs KO sham, **p = 0.0015), 14 d (WT SNI vs WT sham, *p = 0.0022; KO SNI vs KO sham, ****p < 0.0001), 21 d (WT SNI vs WT sham, ****p < 0.0001; KO SNI vs KO sham, ***p = 0.0003), and 28 d (WT SNI vs WT sham, ***p = 0.0002; KO SNI vs KO sham, ****p < 0.0001). There were no statistically significant differences between WT and *Tmem97* KO mice within a treatment (i.e., SNI or sham) at any of the time points. In other words, the loss of *Tmem97* did not disrupt normal SNI-induced changes in pain-like sensitivity (Fig. 8). Finally, we conducted a sensorimotor behavior battery to validate whether anxiety-like and depressive-like behavioral phenotypes observed in these mice were driven by motor deficits induced by genotype and/or neuropathic injury. The sensorimotor battery revealed no signs of apparent ataxia or motor deficits both before and 10 weeks after SNI or sham surgery (Extended Data Fig. 8-1*A–J*).

10.1523/ENEURO.0488-23.2024.f8-1 Figure 8-1 **Sensory-motor behavioral battery performed with wild-type and *Tmem97* KO mice.** No effect of genotype was observed in a battery of sensory-motor behaviors. No significant difference between genotypes is observed in **(*A*)** walk initiation, **(*B, C*)** turn on pole, **(*D*)** ledge time, or **(*E*)** platform time. **(*F* - *I*)** A trend for less latency to reach the bottom or top on a 60° and 90° inclined wire mesh was observed after surgery but there was no effect of genotype. **(*J*)** No significant difference was observed on the inverted wire mesh. [Repeated Measure, Mixed model ANOVA with allowing different variances for genotype and adjusted for Bonferroni method]. Values are mean +/- SEM, Significant difference. Download Figure 8-1, TIF file.

Overall, both WT and *Tmem97* KO mice developed consistent mechanical hypersensitivity following SNI with no genotype-specific variations that were significantly different from the sham group. The sensorimotor behavior assessments revealed no signs of ataxia induced by genotype or neuropathic injury before or 10 weeks after surgery, confirming that the observed affective behavioral changes were not driven by gross motor deficits.

## Discussion

In this study, the loss of *Tmem97* expression was found to be associated with reduced anxiety-like and depressive-like behaviors at baseline in the LDP and TST but not in other affective behaviors. The effect of genotype on anxiety-like and depressive-like behaviors was also influenced by sex, with female *Tmem97* KO mice showing unique patterns of reduced anxiety-like and depressive-like behavior in LDP and TST, respectively. Additionally, the study found that prolonged exposure to neuropathic injury led to *Tmem97*-associated depressive-like behaviors in WT female mice, specifically with the FST. This effect does not seem to be related to expression differences of TMEM97 as no sex differences in expression have been found (Quadir et al., 2021). Future studies can address the mechanism underlying sex-specific *Tmem97*'s role in modulating affective behaviors in the context of neuropathic pain. Overall, these data do show that *Tmem97* has a modest but complex impact on affective behavior under naive and injured conditions.

We have observed that *Tmem97*-associated anxiety-like and depressive-like behavior is dependent on the type of behavior assay being tested. *Tmem97* may regulate anxiety-like and depressive-like behavior in a modality-dependent manner. One possible interpretation is that the behavioral assays in question may differ in the sensitivity to certain genotypes and/or testing environment. Some tests may be more subtle or more robust in detecting changes in behavior, which maybe masked in a certain condition (Ramos, 2008; Guilloux et al., 2011; Rosas-Sánchez et al., 2021). For example, the anxiolytic effect in *Tmem97* KO is only shown in LDP but not in OF, which could have been obscured by the long testing duration in OF. Also, it is important to keep in mind that affective behavioral assays are sensitive to environmental factors and individual variability, such as resilience to stress, which could alter the effect. In the circumstance such as LDP, the initial high activity in the latency to light chamber is due to the increased escape tendency of WT mice in a new environment and not necessarily by the decrease in risk avoidance (Aulich, 1976; La-Vu et al., 2020) due to anxiety. Another possible interpretation is that different behavioral assays model various aspects of anxiety and depression. For example, EPM measures the “fear of height” as anxiety, but LDP measures the “fear of an illuminated open area” as anxiety. These different behavioral assays may engage different neural circuits underlying the anxiety- or depressive-like behaviors. Both FST and TST are typically used to measure depressive-like behaviors by measuring the learned hopelessness as reflected in time immobile. However, the underlying pathophysiology in FST and TST is different (Chatterjee et al., 2012), and this may reflect how *Tmem97* modulates affective behaviors in a context-dependent manner. To help interpret the full phenotype for *Tmem97* KO mice, we pursued a *Z* score process that was developed to determine the overall affective phenotype when individual behaviors did not match (Guilloux et al., 2011; Lax et al., 2018; Bao et al., 2023; Bordes et al., 2023; Castelli et al., 2023; J. Wang et al., 2023; Li et al., 2023). When we evaluated the full affective phenotype or “emotionality,” we found that *Tmem97* disruption decreased anxiety-like and depression-like behavior.

Our findings indicate that mammals would seemingly benefit from the loss of *Tmem97* because it reduces the development of affective comorbidities associated with pain. Because pain condition and affective disorders coexist and worsen symptoms in a reciprocal manner, it is difficult to effectively treat pain comorbidities of affective disorders with conventional approaches (Antioch et al., 2020; Roughan et al., 2021). The shared pathophysiology underlying the two different conditions may contribute to the development of affective comorbidities in pain disorders (C. Han and Pae, 2015; Sheng et al., 2017; Zheng et al., 2022). Studies have suggested that brain regions such as the medial prefrontal, insular, anterior temporal cortices, hypothalamus, hippocampus, and amygdala are known to be associated with regulating pain and emotions at circuitry level (Fields, 2000; Price and Drevets, 2010; Etkin et al., 2011; Zhou et al., 2019; Chen et al., 2022). These brain regions receive projections from pain-modulating brainstem structures, like the periaqueductal gray (PAG) and rostral ventromedial medulla (RVM) via a neural pathway known to underlie pain comorbidities of affective disorders (Gutstein et al., 1998; Fields, 2000; Crawford et al., 2021). A single-cell transcriptomic study found that *Tmem97* is ubiquitously expressed in several brain regions including the hippocampus, substantia nigra, cortex, and cerebellum (Karlsson et al., 2021). In our studies, *Tmem97* is expressed in brain regions like the basal lateral amygdala (BLA), dorsal medial hypothalamus (DMH), and CA3 hippocampal layers of hippocampus that get input from the PAG and RVM (Y. Yang and Wang, 2017; S. Yang and Chang, 2019). Perhaps a *Tmem97* expressing cell-mediated neural pathway that is known to play a role in pain and affective disorders contributes to the neuropathic injury-induced depressive behaviors observed in our studies. Future studies investigating the role of *Tmem97* at the circuitry level or using genetic tools may help predict off-target effects of new s_2_R/TMEM97 ligands that are under development (Ozdas et al., 2020; Ozawa and Arakawa, 2021; J. H. Wang et al., 2023).

In this study, the loss of *Tmem97* demonstrates a protective effect from the development of depressive-like behavior induced by prolonged neuropathic pain in female mice. However, previous studies using pharmacological agents found that modulation of s_2_R reduced anxiety-/depressive-like behaviors in naive rodents through a mechanism interpreted as agonism (Sánchez et al., 1997; Sánchez and Papp, 2000), which seemingly conflicts with our finding since Tmem97 KO mice could be interpreted as equivalent to “antagonistic” modulation of the receptor. This discrepancy may be due to the off-target effects of putative ligands developed before the s_2_R was identified as TMEM97 in 2017 (Alon et al., 2017). That is, the findings from these previous studies may not be due to TMEM97 function and putative ligands developed before 2017 may be targeting other receptors with a binding site similar to that of s_2_R/TMEM97. This is evidenced by the fact that many pharmacological studies prior to 2017 did not validate effects using transgenic models, such as the *Tmem97* global knock-out mice used in the present study (Sánchez et al., 1997; Sánchez and Papp, 2000). Additionally, there is a controversy in the definition of agonist/antagonist modulators of s_2_R/TMEM97 in pain modulation. For example, both “agonists” (Sahn et al., 2017) and “antagonists” (Kotagale et al., 2013) relieve pain-like effects with both ligands showing high affinity to s_2_R/TMEM97.

Ultimately, this may speak to the complexity of s_2_R/TMEM97 function. Activation of *Tmem97* does not necessarily translate into upregulation of *Tmem97* function. This is an unusual protein with no canonical signaling pathway; therefore, the effect of s_2_R/TMEM97 activation does not adhere to a binary paradigm like many other receptors. The receptor's effects are multifaceted and contingent upon the specific biological context in which the receptor is activated or inhibited. This is highlighted by biological pathways known to be associated with s_2_R/TMEM97, which appears to be widespread throughout the nervous system with various functions. s_2_R/TMEM97 interacts with progesterone receptor membrane component 1 (PGRMC1) to modulate autophagy in brain and retinal related neuronal degenerative disease (Ostenfeld et al., 2008; Zeng et al., 2012; Limegrover et al., 2021; Shen et al., 2021). s_2_R/TMEM97 is also involved in regulating cholesterol homeostasis by facilitating low-density lipoprotein receptor (LDLR) internalization at the plasma membrane and forming intracellular complexes in Niemann–Pick disease, type C1 (NPC1; Bartz et al., 2009; Ebrahimi-Fakhari et al., 2016; Riad et al., 2018, 2020). In earlier studies, s_2_R/TMEM97 in the rodent brain was investigated as a haloperidol-sensitive receptor that plays a role in modulating the intracellular phosphoinositide-associated signaling pathway (Bowen et al., 1988a,b). Later, the same group of scientists found that s_2_R/TMEM97 in the brain mediates antipsychotic drug-induced motor dysfunction (Walker et al., 1988; Bowen et al., 1988b; Goldstein et al., 1989); this highlights the need to investigate behavioral change as a result of modulating s_2_R/TMEM97 in the central nervous system. In an oncogenic context, s_2_R/TMEM97 is implicated in the *Wnt*/*beta*-catenin pathway and upregulates low-density lipoprotein receptor related protein 6 (LRP6) phosphorylation that ultimately results in tumor growth (Pai et al., 2017). Previous literature describing the impact of s_2_R/TMEM97 on disease is highly dependent on the involved pathway and not necessarily the simple presence of the receptor.

In our study, *Tmem97* genetic disruption does not directly modulate mechanical hypersensitivity after SNI. This finding is consistent with three other studies that have shown that activation of s_2_R/TMEM97 pharmacologically temporarily reduces SNI-induced mechanical hypersensitivity (Sahn et al., 2017; Alon et al., 2021; Yousuf et al., 2023). We recently reported in a separate study no effect of *Tmem97* disruption on neuropathic injury-induced mechanical hypersensitivity (Yousuf et al., 2023). In that study, WT and Tmem97 KO mice in both sexes were indistinguishable at baseline and for 2 weeks after SNI injury. The present study, we extend these findings now out to 4 weeks postinjury. In the context of *Tmem97* disruption, we might, in fact, anticipate more hypersensitivity in the *Tmem97* KO mice. Both WT and *Tmem97* KO mice are at the “floor” of the mechanical sensitivity assay after SNI preventing exploration of this hypothesis. Future studies with reversable or submaximal injuries could be used to further explore these questions.

Chronic neuropathic pain not only influences affective behaviors but is often also associated with cognitive deficits (Moriarty et al., 2017; Khera and Rangasamy, 2021). Cognition and mood are domains that are strongly tied to each other in modifying affective behaviors and learning performance in rodents (Petković and Chaudhury, 2022), especially under long-term exposure to pain. It is important to investigate whether *Tmem97* is associated with cognitive domains to separate the effect of genotype observed in baseline affective behaviors. One study found that treating mice with DKR-1677, a s_2_R/TMEM97 modulator, rescued a traumatic brain injury-induced impairment in spatial learning and memory in mice and prevented neuronal cell loss in the hippocampus (Vázquez-Rosa et al., 2019). In our set of cognitive function assays, however, we failed to find a difference between genotypes and sex under naive conditions suggesting that executive function is largely intact in the *Tmem97* KO mice. The results of the two types of behavioral batteries together suggest that *Tmem97*-associated modulatory role is specific to anxiety-like and depressive-like behaviors and there is no overt executive function-related behavioral confounding this interpretation.

There is limited literature regarding the role of *Tmem97* in developmental biology. Given the use of conventional *Tmem97* KO mice in the current study, it is possible that some effects were driven by *Tmem97* impacts on neural development rather than adult animal *Tmem97* function. It is well understood that in global KO mice, broadly speaking, developmental changes can potentially mask the function of the investigated gene in the adult state (Walrath et al., 2010). However, based on current literature, *Tmem97* is not one of critical genes that regulate pattern formation and cell fate programming during embryonic development (Alon et al., 2017). *Tmem97* KO mice show no overt phenotype. They breed normally and are of normal weight. We found no obvious sensory or motoric deficits in these mice. Future studies focused on the developmental role of *Tmem97* would be valuable. s_2_R/TMEM97 is strongly associated with cell proliferation in cancer (Qiu et al., 2015) and cell proliferation is obviously critical to development (Matson and Cook, 2017). Our results also point to a broader question of whether having *Tmem97*-regulated heightened anxiety-/depressive-like traits is evolutionarily advantageous for individual fitness (Raison and Miller, 2013; Walters and Williams, 2019). Studying the role of *Tmem97* in developmental biology would provide new insight to what benefit an intact organism might have in gaining function of *Tmem97* from an evo-devo perspective.

Prolonged exposure to neuropathic injury with pain is associated with anxiety-like and depressive-like behaviors in animal models (Dimitrov et al., 2014; Lax et al., 2014; Sieberg et al., 2018). Previous pharmacological studies focused on the effect of putative ligands on affective or pain behaviors (Sánchez et al., 1997; Sánchez and Papp, 2000; Kotagale et al., 2013; Sahn et al., 2017; Vázquez-Rosa et al., 2019; Intagliata et al., 2020; Alon et al., 2021; Quadir et al., 2021; Shen et al., 2021) but did not study how *Tmem97* directly plays a role in the comorbidity relationship. Our study focused primarily on the gene, *Tmem97*, to characterize its effect in various affective behaviors and to what extent it changes postoperative anxiety-like and depressive-like behaviors after exposing to prolonged neuropathic injury. The study revealed that the loss of *Tmem97* expression was linked to reduced anxiety-like and depressive-like behaviors in specific assays largely in female mice only. We also found that prolonged exposure to neuropathic injury is associated with depressive-like behaviors only in WT female mice, indicating that *Tmem97* has a modest yet complex impact on affective behavior under neuropathic pain conditions. The biological function of s_2_R/TMEM97 needs to be further investigated to better understand its potential role as a therapeutic target to treat pain, affective disorders, or affective comorbidities of pain disorders.

## Synthesis

Reviewing Editor: Jonathan Lee, University of Birmingham Decisions are customarily a result of the Reviewing Editor and the peer reviewers coming together and discussing their recommendations until a consensus is reached. When revisions are invited, a fact-based synthesis statement explaining their decision and outlining what is needed to prepare a revision will be listed below. The following reviewer(s) agreed to reveal their identity: Chandrashekhar Borkar, Gessynger Morais-Silva.

The authors have fully addressed all of the previous reviewer comments. We commend the constructive approach to the revisions.
