# Supplementary figures and images for: Loss of Sigma-2 Receptor/TMEM97 Is Associated with Neuropathic Injury-Induced Depression-Like Behaviors in Female Mice
Source: eNeuro. 2024 Jun 28;11(7):ENEURO.0488-23.2024. doi: 10.1523/ENEURO.0488-23.2024 (PMC11228697; doi:10.1523/ENEURO.0488-23.2024)

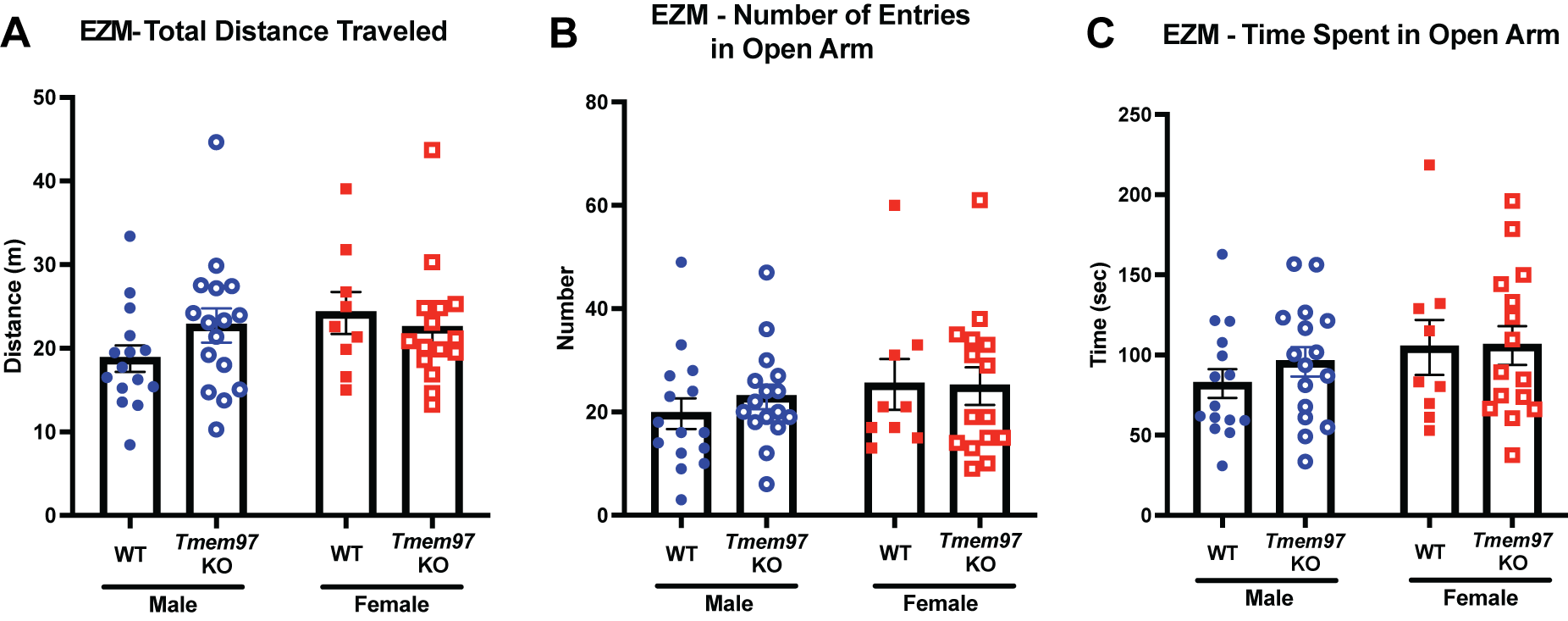

Supplement: Figure 3-1 — No significant difference between anxiety-like behaviors between wild-type and Tmem97 KO mice in both sexes using elevated zero maze. (A, B, C) In naïve mice, no significant difference between genotypes in both sexes is observed in any parameter of the EZM: total distance traveled, number of entries in open arm, and time spent in open arm. EZM = elevated zero maze. [Two-way ANOVA with Tukey's multiple comparison test]. Values are mean +/- SEM. Download Figure 3-1, TIF file. [file eneuro-11-ENEURO.0488-23.2024-s002.tif]

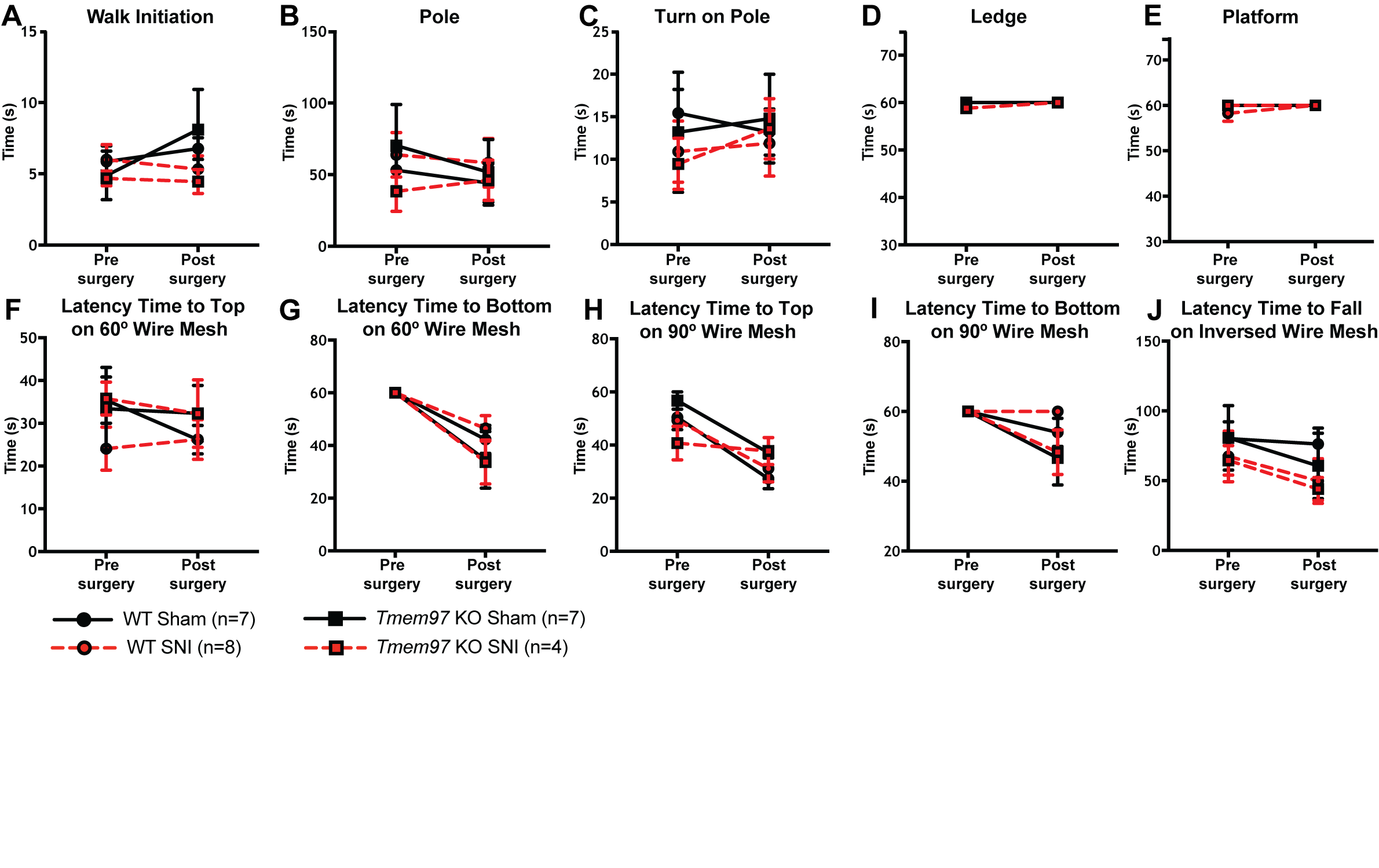

Supplement: Figure 8-1 — Sensory-motor behavioral battery performed with wild-type and Tmem97 KO mice. No effect of genotype was observed in a battery of sensory-motor behaviors. No significant difference between genotypes is observed in (A) walk initiation, (B, C) turn on pole, (D) ledge time, or (E) platform time. (F - I) A trend for less latency to reach the bottom or top on a 60° and 90° inclined wire mesh was observed after surgery but there was no effect of genotype. (J) No significant difference was observed on the inverted wire mesh. [Repeated Measure, Mixed model ANOVA with allowing different variances for genotype and adjusted for Bonferroni method]. Values are mean +/- SEM, Significant difference. Download Figure 8-1, TIF file. [file eneuro-11-ENEURO.0488-23.2024-s003.tif]
